# Metatranscriptomics analysis reveals the cotton virome in the southern United States

**DOI:** 10.1038/s41598-026-40828-5

**Published:** 2026-02-23

**Authors:** Cesar Escalante, Anyi M. Reyes, Chaoyang Zhao, Kipling S. Balkcom, Alana L. Jacobson, Amanda Strayer-Scherer, Kathleen M. Martin, Jenny Koebernick, Anders Huseth, Edmund Kozieł, Ian Small, Jeremy K. Greene, Katarzyna Otulak-Kozieł, Michael J. Mulvaney, Paul P. Price, Ricardo I. Alcalá Briseño, Sudeep Bag, Kassie Conner

**Affiliations:** 1 Present Address: Department of Botany and Plant Pathology, Purdue University, West Lafayette, 47907 IN USA; 2 Department of Entomology and Plant Pathology, Auburn University, Auburn, 36849 AL USA; 3 National Soil Dynamics Laboratory, USDA-ARS, Auburn, 36849 AL USA; 4 Department of Crop, Soil and Environmental Sciences, Auburn University, Auburn, 36849 AL USA; 5 Department of Entomology, Michigan State University, East Lansing, 48824 MI USA; 6 Department of Entomology and Plant Pathology, North Carolina State University, Raleigh, NC 27695 USA; 7 Institute of Biology, Department of Botany and Plant Physiology, Warsaw University of Life Sciences-SGGW, 159 Nowoursynowska Street, Warsaw, 02-776 Poland; 8 Department of Plant Pathology, University of Florida, Quincy, 32351 FL USA; 9 Department of Plant and Environmental Sciences, Edisto Research and Education Center, Clemson University, Blackville, 29817 SC USA; 10 Department of Plant and Soil Sciences, Mississippi State University, Mississippi State, 39762 MS USA; 11 Department of Plant Pathology and Crop Physiology, Macon Ridge Research Station, LSU AgCenter, Winnsboro, 71295 LA USA; 12 Department of Plant Pathology and Environmental Microbiology, Pennsylvania State University, University Park, 16802 PA USA; 13 Department of Plant Pathology, University of Georgia, Tifton, 31794 GA USA; 14 Alabama Cooperative Extension System, Auburn University, Auburn, 36849 AL USA

**Keywords:** Biotechnology, Genetics, Microbiology, Molecular biology, Plant sciences

## Abstract

High-throughput sequencing (HTS) has expanded our perspective on the distribution and diversity of plant viruses. Furthermore, improvements in HTS and decreasing sample costs have enabled the discovery of novel plant viruses in field-collected samples. This study examined the putative virome of cotton samples collected from fields across the southern United States. Leaf samples were collected, and total RNA was extracted. Library preparation was performed from pooled samples within locations before sequencing on an Illumina platform. Sequenced libraries were mapped to the cotton reference genome, and the resulting sequences were *de novo* assembled. A metatranscriptomics analysis revealed complete genome contigs of cotton leafroll dwarf virus in all tested samples. Additionally, 29 putative families of RNA and DNA plant viruses co-infecting cotton were found. Seven families of RNA viruses were more prevalent across all locations. These families included *Botourmiaviridae*, *Hypoviridae*, *Mitoviridae*, *Narnaviridae*, *Partitiviridae*, *Solemoviridae*, and *Totiviridae*. The information obtained in this investigation will help develop a broader perspective on cotton virus diversity and whether co-infections of viruses can influence (negatively or positively) plant physiology, product quality, and yield.

**Supplementary Information:**

The online version contains supplementary material available at 10.1038/s41598-026-40828-5.

## Introduction

Upland cotton (*Gossypium hirsutum*) is the world’s most important fiber for manufacturing clothing and various textiles, and the United States is among the largest cotton producers in the world, representing approximately 11% of global production^1^. Most of the cotton is produced in the southern United States^2^. In 2017, cotton plants displaying virus-like symptoms were observed in several fields in Alabama, and later identified as cotton leafroll dwarf virus (CLRDV)^3^. Subsequently, CLRDV was found infecting cotton across all cotton-growing regions of the United States^4–16^.

CLRDV has been associated with a wide variety of symptoms, including rugosity, stunting, leaf cupping, leaf bronzing, petiole reddening, and wilting^17^. These observed symptoms agree with isolates and disease symptoms specifically reported in South America and Asia^17,18^. However, the presence of symptoms does not always correlate with the presence or absence of CLRDV in the host based on molecular tests for the CLRDV isolate from the United States^14,19^. Therefore, symptoms caused by CLRDV need to be better characterized to accurately diagnose putative viral infections from field sample collections. There could be several reasons causing this inconsistency in symptom expression versus the presence of viral agents in a plant. For example, a plant can have a wide spectrum of phenotypic responses under various abiotic factors, such as temperature extremes and light intensity^20^. Additionally, mixed viral infections can cause synergistic or antagonistic interactions in economically important crops^21,22^. Viruses across different families can produce similar symptoms, while others may be asymptomatic. This makes virus symptoms and genome characterization difficult; therefore, using advanced, up-to-date technologies such as High throughput sequencing (HTS) in combination with biological experiments (such as virus indexing), will make this task more feasible.

Next-generation sequencing or HTS has transformed how we detect and identify plant viruses because it can eliminate the need for prior knowledge of their presence in the host^23,24^. This method has been applied to sequence genomes of new viruses in crops and wild plants, which may serve as hosts for plant viruses^25–27^. The identification of novel pathogens is crucial to make disease management more efficient by considering the role of multiple pathogens during molecular or other types of diagnostic data analysis methods, such as network analysis^28,29^. The overall goal of this study was to implement a metatranscriptomics approach to understand the regional distribution of the cotton plant virome from samples collected across several states of the Cotton Belt. Information generated in this study will enhance knowledge about virus diversity in cotton and whether co-infections of viruses can influence (negatively or positively) plant physiology, product quality, and yield.

## Results and discussion

### RNA sequencing data

Ten libraries were sequenced, yielding over 100 million reads each. On average, 51.6 million clean reads were retained after quality assessment and Illumina adapters removal, ranging from 42.7 to 71.1 million (Table 1). Most of the libraries showed a host alignment rate higher than 70%. The Quincy, FL, and Winnsboro, LA libraries showed low alignment rates of 25.5 and 39.2%, respectively; likely due to sequence quality and overall higher repetitive regions of the two libraries. The GC content ranged from 43.2 to 52.4% across the 10 libraries (Table 1).

**Table 1:** Summary statistics of RNA-sequencing from cotton samples collected across the southern United States.

State/Location	Raw reads	Clean reads^a^	Alignment rate (%)^b^	GC (%)	Accession
Alabama-Fairhope	96,204,652	48,018,696	86.35	47.13	SAMN37366055
Alabama-Headland	99,770,042	49,754,024	75.65	46.98	SAMN37366056
Alabama-Prattville	97,584,270	48,657,117	77.23	48.68	SAMN37366057
Alabama-Brewton	90,934,744	45,351,806	73.70	45.53	SAMN37366058
Florida-Jay	94,083,346	46,920,273	76.25	46.14	SAMN37366059
Florida-Quincy	132,597,656	66,120,371	25.54	52.36	SAMN37366060
Georgia-Tifton	86,046,322	42,741,331	83.87	45.72	SAMN37366061
Louisiana-Winnsboro	142,520,326	71,114,831	39.25	50.40	SAMN37366062
North Carolina-Jackson Springs	103,477,272	51,643,688	82.57	45.83	SAMN37366063
South Carolina-Blackville	91,549,974	45,903,366	81.11	43.22	SAMN37366064
Average	103,476,860	51,622,550	70.15	47.20	N/A
Total	1,034,768,604	516,225,503	N/A	N/A	N/A
Minimum	86,046,322	42,741,331	25.54	43.22	N/A
Maximum	142,520,326	71,114,831	86.35	52.36	N/A

N/A= not applicable.

^a^Number of clean reads after trimming sequence ends using Trimmomatic software.

^b^Overall alignment rate of clean reads mapped to the cotton reference genome using Bowtie2 software.

### Putative viral contigs

**Fig. 1:**
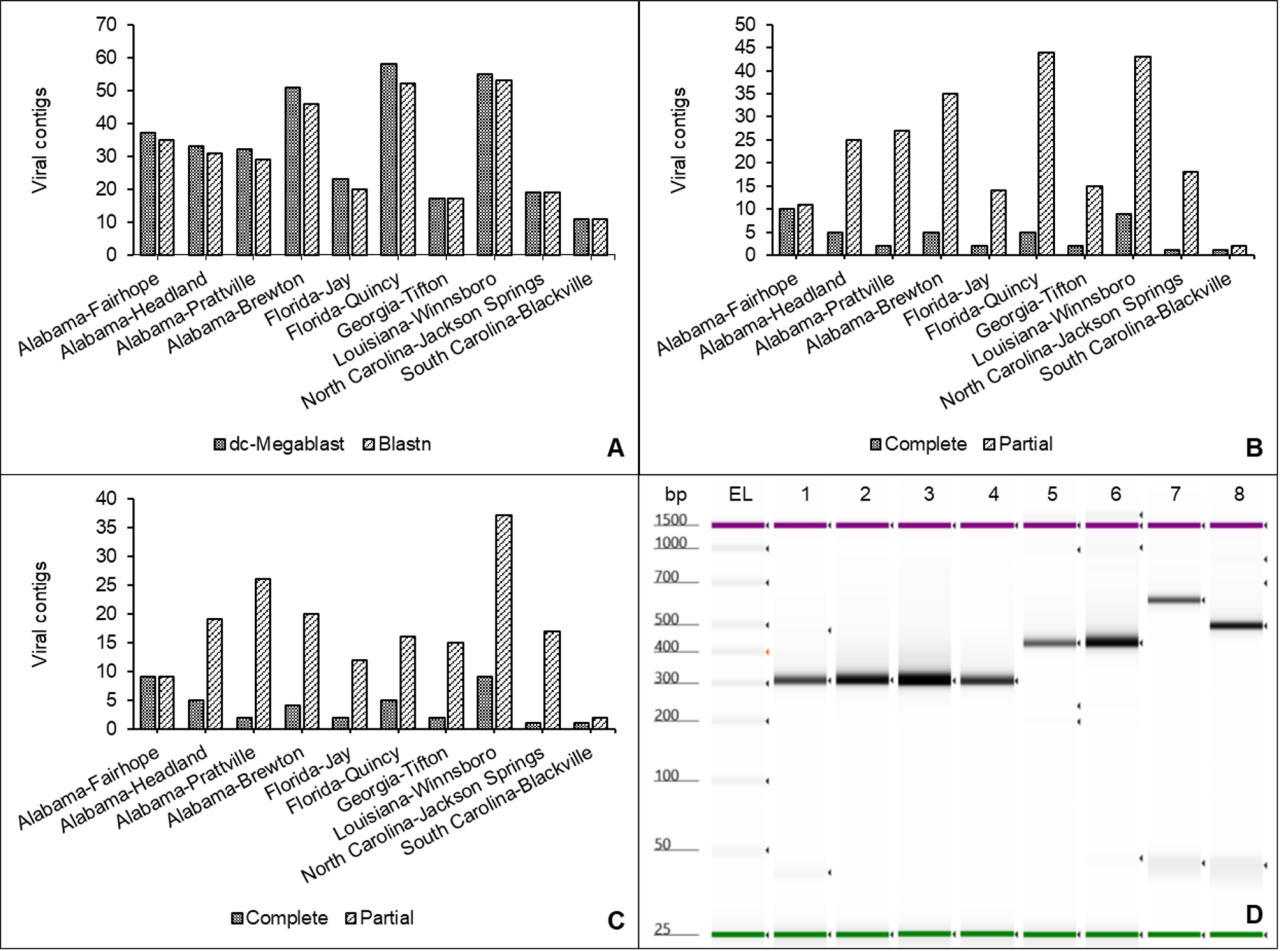
Summary statistics of putative viral contigs obtained from cotton samples collected across the southern United States and PCR products obtained from selected sequences. **(A)** comparison of total viral contigs obtained from a discontiguous megablast (dc-megablast, *n* = 336 contigs) versus Blastn search (*n* = 313 contigs). Bacteriophage sequences were included in this analysis. **(B)** Complete versus partial viral contigs. Sequence completeness was assigned with NCBI Virus database. Bacteriophage sequences were excluded from this analysis. **(C)** Complete versus partial viral contigs using only sequences higher than 70% query coverage. Bacteriophage sequences were excluded from this analysis. **(D)** PCR products from selected viral sequences; lanes bp and EL= base pairs of the electronic ladder, lanes 1–4 = cotton leafroll dwarf virus (*Solemoviridae*) from Fairhope, Alabama; Headland, Alabama; Prattville, Alabama; and Brewton, Alabama, respectively, lanes 5–6 = soybean leaf-associated mitovirus 2 (*Mitoviridae*) from Prattville, Alabama and Quincy, Florida, respectively, lane 7 = plant associated botourmia-like virus 1 (*Botourmiaviridae*) from Fairhope, Alabama, and lane 8 = grapevine-associated levi-like virus 10 (*Fiersviridae*) from Brewton, Alabama. Results from grapevine partitivirus are not included due to amplification and Sanger sequencing inconsistencies.

Of the 10 libraries analyzed, seven were originated from pooled samples, while three (Quincy, FL; Winnsboro, LA; and Jackson Springs, NC) were derived from individual plant samples (Supplementary Table 1). Initial filtering of putative viral sequences was performed with the Galaxy platform using the dc-megablast, producing 336 contigs. The second iteration using blastn and the low complexity algorithm yielded 313 contigs (Fig. 1A). Sequences with homology to plants and other non-plant virus sequences were removed from the analyses. A total of 276 contigs were homologous to viruses, 42 contigs were sequences with lengths similar to complete viral genomes, and 234 contigs were partial sequences (Fig. 1B). Locations in Fairhope, AL, and Winnsboro, LA showed the highest number of complete viral sequences with 10 and 9 contigs, respectively. Locations in Quincy, FL, Winnsboro, LA, and Brewton, AL, had the highest number of partial putative viral sequences with 44, 43, and 35 contigs, respectively (Fig. 1B). Blackville, SC showed the overall lowest number of complete and partial putative viral sequences. Using sequences with 70% or more query cover reduced the overall number of putative viral sequences across all locations, except for Winnsboro, LA, which maintained a similar number of complete and partial putative viral sequences regardless of the query coverage discrimination value used the analysis (Figs. 1B-C).

### Diversity and frequency of virus sequences across locations

**Fig. 2:**
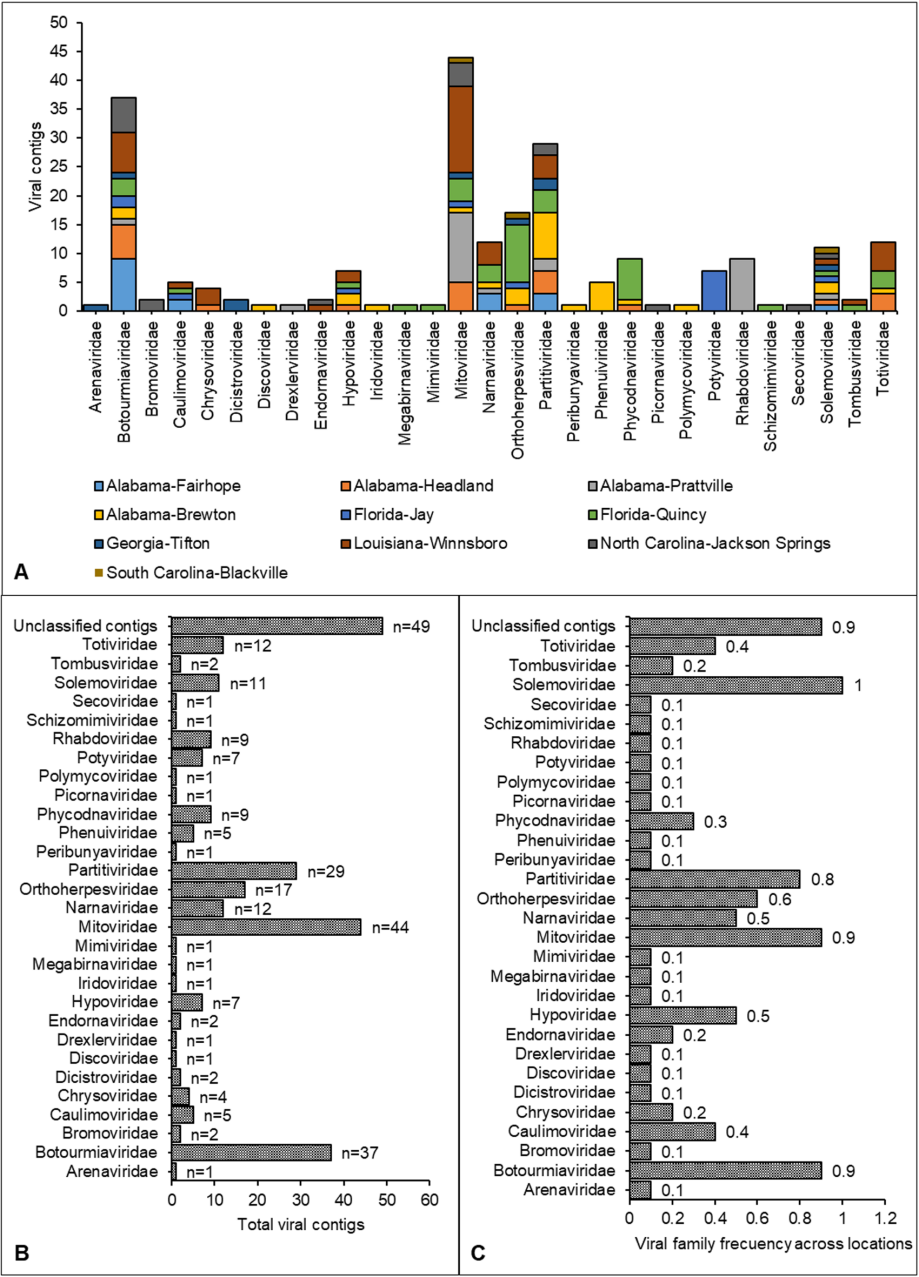
Abundance of putative viral contigs per family. **(A)** Number of viral contig per family across 10 locations in the southern United States. **(B)** List of viral families and contigs across locations included in this study. **(C)** Viral family frequency across locations included in this study. Sequences with homology to bacteriophages, amoebas, etc., were excluded from these analyses.

After taxonomic classification at the family level, 29 viral families were identified across the 10 locations in the southeastern United States (Fig. 2). Some of these contigs were partial sequences classified to the genus and species level (Supplementary Tables 2–11). However, in this study, no major inferences were made on putative viral sequences at the genus and species level because further analyses would be needed to make any conclusions based on molecular composition and biological properties of the putative viral sequences. A more meticulous study would be required to determine which contigs potentially belong to novel or the same viral species. Among the 29 putative viral families identified, seven families of RNA type were more prevalent across all locations with more information for each family provided.

#### Botourmiaviridae

Except for Blackville, SC, contigs of this family were found across all locations tested (Fig. 2A). Samples from Fairhope, AL, contained the highest number of assembled contigs. Four genera are included in this family, and *Ourmiavirus* is the genus with species that infect plants^49^. The genome of botourmiaviruses is relatively small, ranging from 2 to 3 kb. Plant-infecting botourmiaviruses are multipartite and the rest (mycoviruses) are monopartite^49^. In this study, several contigs were tagged as partial; however, these may represent segments of multipartite genomes that together constitute complete ourmiavirus genomes. Further analysis is required to confirm this viral genomic structure. Similarly, as observed in the family *Botourmiaviridae*, several partial contigs from other viral families identified in this study may also correspond to complete genomes. However, additional studies are necessary to assess the completeness of these putative viral sequences. Virus species in the genus *Ourmiavirus* have shown a wide variety of symptoms in plants that include chlorotic spots, ringspots, yellow mosaic, and stunting^49,50^. When samples used for this study were collected, a comprehensive number of symptoms were recorded; however, most of the symptoms were associated with the presence of CLRDV, which mainly causes leaf deformation and plant stunting. As documented in several cases, it is difficult to discriminate symptom patterns in plants infected by multiple viruses^51^. Therefore, isolation and purification of individual viral organisms is needed to characterize symptom patterns and expression caused by single or mixed viral infections in cotton.

#### Hypoviridae

Seven contigs of hypovirids were found in five locations, which included libraries from Alabama, Florida, and Louisiana (Fig. 2, Supplementary Tables 3, 5–7, and 9). Hypovirids have mainly been found in fungi. However, viruses of this family have putatively also been found in arthropods, such as insects^52^. Only partial sequences of putative members of this family were detected in our analysis. These organisms could be in low quantities within the tissue analyzed; therefore, the depth of sequencing used in this study would not amplify or recover sequences from low concentrations of viral RNA. Viral species of this family are known to be hypovirulent and reduce the disease caused by certain fungal pathogens^53^. A typical example of hypovirids causing hypovirulence is Cryphonectria hypovirus 1, which infects *Cryphonectria parisitica*, the fungus that devasted American chestnut trees (*Castanea dentata*) in the United States^53–55^. Isolation of known and unknown fungal pathogens that host hypoviruses and perhaps cotton endophytes, could help determine the role of these viruses in the cotton microbiome and explore the potential use of hypoviruses in management of cotton fungal diseases.

#### Mitoviridae

Members of this family consisted of 44 putative viral contigs assembled in this study; they were found across nine locations (Fig. 2A-B, and Supplementary Tables 3–11). The library from Fairhope, AL, was the only one without *Mitoviridae* contigs (Fig. 2A). Members of the family *Mitoviridae* are hosted by fungi, and one of the reference species is Cryphonectria parasitica mitovirus 1, hosted by *C*. *parisitica*^56,57^. Like hypovirids, species in this family have been studied for their potential as biological control agents of several fungal pathogens^58^.

#### Narnaviridae

Twelve contigs consisting of nearly complete sequences of putative narnaviruses were obtained from five locations in Alabama, Florida, and Louisiana (Fig. 2, Supplementary Tables 2, 4, 5, 7 and 9). Members of this family infect filamentous fungi, yeast and oomycetes^59^. Genomes of these viruses consist of only one open reading frame encoding for the RdRp domain. Their genome size ranges from 2.3 to 3.6 kb, and, for their relatively small genome size and protein-coding simplicity, they are considered one of the simplest viral genomes^59^. Two genera were classified in this family, the genus *Mitovirus*, which was initially found only in filamentous fungi, and the genus *Narnavirus*, which is thought to have members that infect yeast and oomycetes^59,60^. Most putative viral sequences of the family *Narnaviridae* found in this study were possibly classified in the genus *Narnavirus*; however, the blast search performed in this study suggests that members of this genus could infect yeast and oomycetes, as well as fungi.

#### Partitiviridae

Putative sequences of members of this family were found in all locations of Alabama, Florida, Georgia, Louisiana, and North Carolina samples (Fig. 2, and Supplementary Tables 2–5, and 7–10). A total of 29 partial sequences were assembled across locations (Fig. 2B). Partitiviruses are commonly found in plants, fungi, and protozoa^61^. In plants, partitiviruses have been described in several economically important crops, including sugar beet (*Beta vulgaris*), pepper (*Capsicum* spp.), and clover (*Trifolium* spp.)^62–64^. Samples collected from Florida, Georgia, and North Carolina consisted of putative members of plant viruses (Supplementary Tables 7, 8, and 10). The remaining putative sequences were likely mycoviruses of this family, presumably from fungal endophytes colonizing cotton samples used in this study. In most cases, plant partitiviruses have not been associated with symptoms, rather, they have been classified in a symptomless category of viruses called persistent viruses^65^. However, a partitivirus, Sclerotinia sclerotiorum partitivirus 1 found in *Sclerotinia sclerotiorum* and *Botrytis cinerea* was associated with hypovirulence in these fungi^66^. As suggested with members of the family *Hypoviridae*, classification of fungal isolates containing partitiviruses will be helpful to understand the tripartite interaction of virus-fungus-plant and its potential use to attenuate the disease caused by its fungal host.

#### Solemoviridae

Putative sequences of CLRDV, a member of the *Solemoviridae* family were found in all samples tested in this study. A total of 11 contigs were assembled, from which, 10 were nearly complete sequences (Fig. 2, and Supplementary Tables 2–11). The family *Solemoviridae* contains members that infect only plants, and they are transmitted by mechanical wounds, vegetative propagations, while many are also transmitted via insects, specifically aphids^67^. CLRDV, a member of this family in the genus *Polerovirus*, is considered an emerging viral pathogen in the United States. The presence of the pathogen has been confirmed in almost every state where cotton is grown^4–16^. Samples selected for this study were previously tested for CLRDV using RT-PCR^14^; therefore, we considered CLRDV an internal control and hypothesized that partial or full contigs could be obtained. By obtaining nearly complete sequences of CLRDV in this study, we confirmed that our sequence pipeline from sample preparation to bioinformatics analysis was robust and met basic requirements to perform metatranscriptomics analysis.

#### Totiviridae

Twelve contigs of putative members of this family were assembled from four locations across three states (Alabama, Florida, and Louisiana) (Fig. 2, and Supplementary Tables 3, 5, 7, and 9). Members of this family are hosted by protozoa and fungi^68^. Several putative viral sequences were partially classified in the genus *Victorivirus*, which has viral species hosted by fungi (Supplementary Tables 3 and 9)^69^. However, several other sequences suspected to be closely related to insect viruses did not have an assigned genus classification (Supplementary Tables 3, 5, 7, and 9). The continued use of HTS has expanded the discovery of viral sequence members of this family that are not only hosted by protozoa and fungi, as suggested by Wickner et al. (2009) in their ICTV taxonomy report. Therefore, by performing biological and molecular analyses, virologists found that members of the family *Totiviridae* have a broad host range, and are also hosted by insects^70,71^, plants^72^, crustaceans, fish and bats^73,74^. These are examples of the efficiency from a metatranscriptomics analysis to study viral diversity and host range; both fundamental to improve diagnostic tools for prompt management, specifically for viruses that could cause major negative effects on crop production.

### Data validation by RT-PCR

Selected sequences for RT-PCR validation consisted of putative plant and mycoviral sequences (Supplementary Tables 12 and 13). Sanger sequences obtained from PCR products were subjected to a blastn search for provisional virus species comparisons. Results from the blastn search showed that sequences shared high nucleotide percent identity to CLRDV (*Solemoviridae*), soybean leaf-associated mitovirus 2 (*Mitoviridae*), plant associated botourmia-like virus 1 (*Botourmiaviridae*), and grapevine-associated levi-like virus 10 (*Fiersviridae*) (Fig. 1D). For sequences from grapevine partitivirus, we obtained amplicons of the expected target size in four locations; however, amplicons showed some inconsistencies and Sanger sequencing did not yield readable chromatograms (Supplementary Table 13). Interestingly, only those sequences related to plant viruses yielded PCR products of the expected size (Fig. 1D and Supplementary Table 13). As previously mentioned, it is possible that some mycovirus-related sequences found in this study could have come from fungal species colonizing the surface of plant tissue. Therefore, the amount of fungal tissue was much less than plant tissues, thus, yielding a lower amount of total RNA. This aspect provides a low concentration RNA template for the putative viral sequences selected, rendering negative PCR results. Furthermore, the current detection rate could potentially be improved by further optimization of the PCR thermal cycling conditions, or the design of alternative primer sets to account for potential viral variants.

### Phylogenetic analyses

**Fig. 3:**
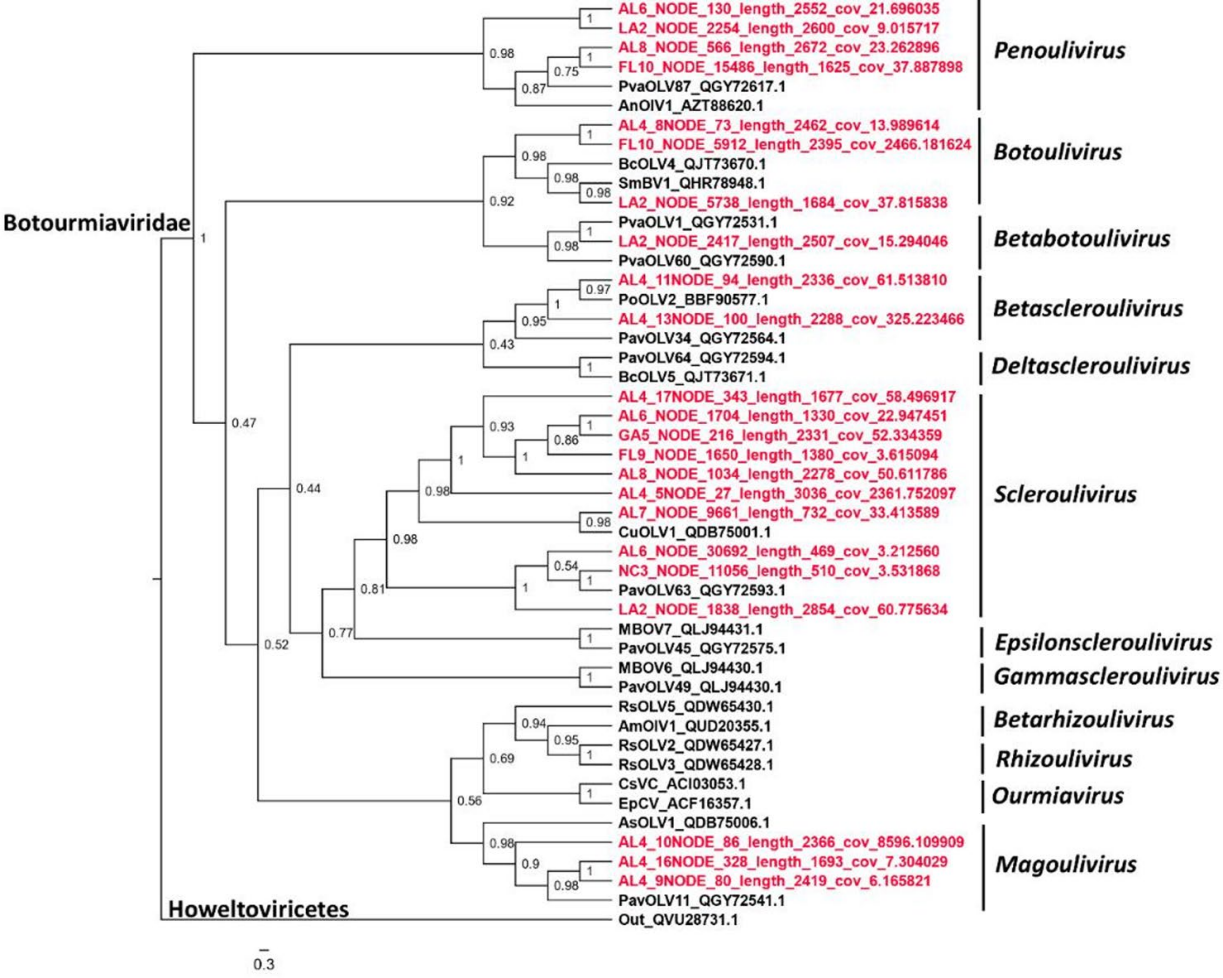
Phylogenetics analysis of putative viral sequences belonging to the family *Botourmiaviridae*. Amino acid sequence of RdRp region from putative viral sequences found in this study (red) were compared to sequences of all 12 genera in the family *Botourmiaviridae*. The tree was rooted on a virus belonging to a different class, *Howeltoviricetes*. Clade posterior probabilities are indicated at nodes. A scale bar at the bottom indicates branch lengths measured in the number of substitutions per site. Sequences from this study are available in GitHub (see data available statement). Accession numbers for sequences publicly available are shown in the tree after the virus’ acronym.

**Fig. 4:**
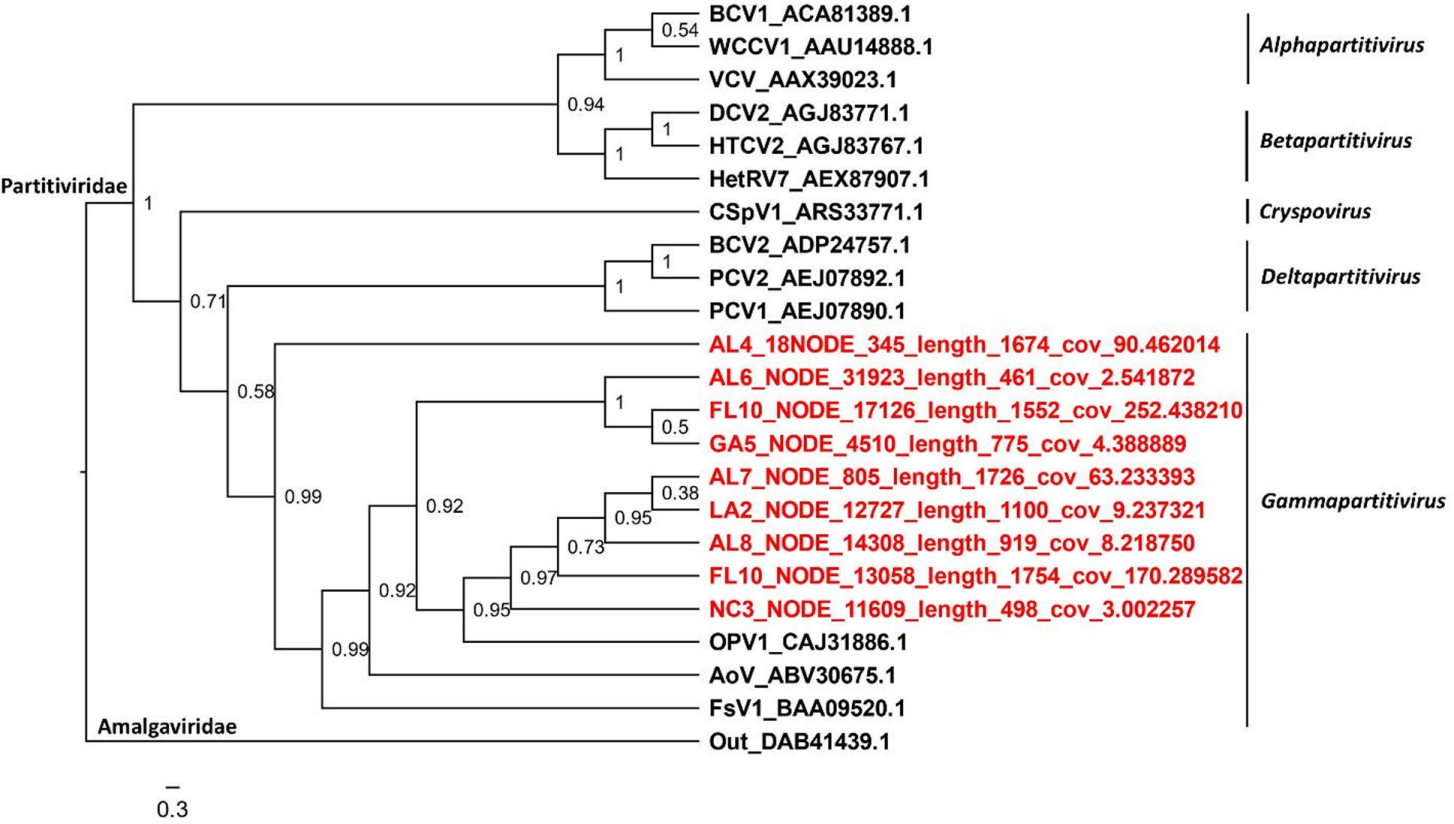
Phylogenetics analysis of putative viral sequences belonging to the family *Partitiviridae*. Amino acid sequence of RdRp region from putative viral sequences found in this study (red) were compared to sequences of all five genera in the family *Partitiviridae*. The tree was rooted on a virus belonging to the family *Amalgaviridae*. Clade posterior probabilities are indicated at nodes. A scale bar at the bottom indicates branch lengths measured in number of substitutions per site. Sequences from this study are available in GitHub (see data available statement). Accession numbers for sequences publicly available are shown in the tree after the virus’ acronym.

**Fig. 5:**
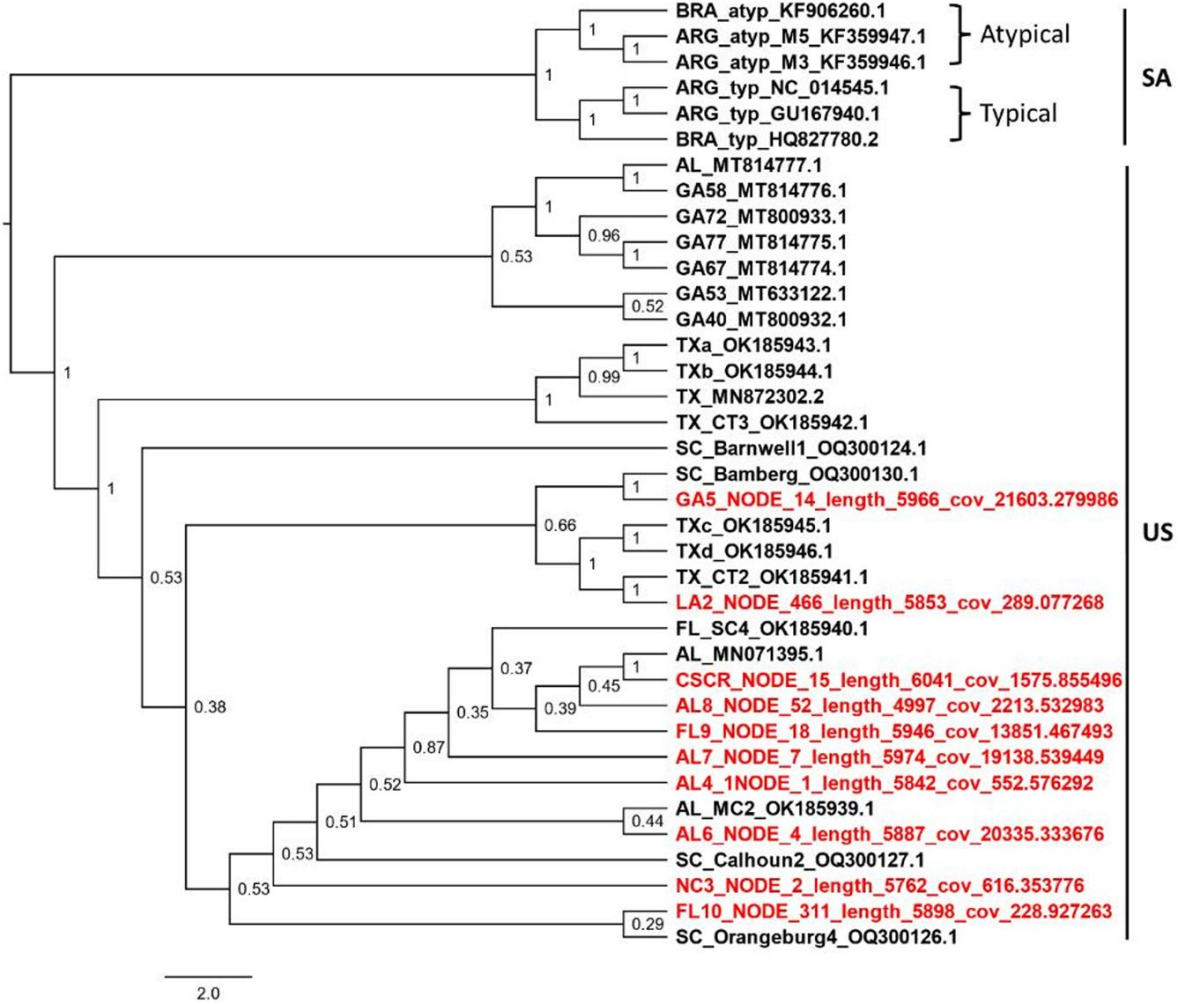
Phylogenetics analysis of putative viral sequences belonging to the family *Solemoviridae*. Amino acid sequence of complete genomes of cotton leafroll dwarf virus (CLRDV) found in this study (red) were compared to sequences of several CLRDV isolates from South America (SA) and the United States (US). The tree was midpoint-rooted. Clade posterior probabilities are indicated at nodes. A scale bar at the bottom indicates branch lengths measured in the number of substitutions per site. Sequences from this study are available in GitHub (see data available statement). Accession numbers for sequences publicly available are shown in the tree.

Putative members of the family *Botourmiaviridae* used in phylogenetic analysis were found in six out of the 12 reported genera (Fig. 3)^49^; they included putative members of the genera *Penoulivirus*, *Botoulivirus*, *Betabotoulivirus*, *Betascleroulivirus*, *Scleroulivirus*, and *Magoulivirus* (Fig. 3). All the aforementioned genera contain viral species hosted by fungi. Interestingly, based on the phylogenetic analysis, plant botourmiaviruses, usually of the genus *Ourmiavirus*, were not observed; however, a more thorough analysis could confirm which putative botourmiaviruses found in this study, are hosted by fungi and plants. Although ourmiaviruses consist of an exclusive group of plant viruses^75^, it is possible that members of other genera could be hosted across kingdoms, including plants. The *Partitiviridae* family comprises five genera^61^. Phylogenetic analysis indicated that the sequences generated from the collected samples were all clustered in the genus *Gammapartitivirus* (Fig. 4). Members of this genus are only found in fungi^61^. This indicates that putative sequences included in this analysis corresponded to fungi colonizing plant tissue in this study. The 10 nearly complete sequences of CLRDV assembled were used for a phylogenetic analysis comparison with other sequences from the United States and South America (Fig. 5). In agreement with several previous studies^76–80^, our analysis indicated that all United States isolates form a single clade (the United States clade) with strong support (posterior probability = 1), separating them from the South America clade, which includes both typical and atypical genotypes of CLRDV (Fig. 5). This phylogenetic relationship highlights molecular composition differences between the United States and South America clades. We hypothesize that these differences influence plant infection responses, particularly symptom expression. Notably, it has been suggested that South American strains are more severe than CLRDV strains from the United States^81^. Although several subclades were observed within the United States clade, samples previously collected from different states and in this study were interlaced, showing no clear geographic patterns.

Information obtained in this investigation expanded our understanding of plant virus diversity in cotton agroecosystems. Many putative viral sequences assembled in this study have been previously reported in other phyla or crops. However, some of these viruses have not been reported in cotton. This metatranscriptomics analysis provided a catalog of putative viruses that may play an important role in the physiological interaction of cotton with other microorganisms. Continued use of HTS technologies has expanded the discovery of viral sequences and their host range. Therefore, findings of previous virome studies and results obtained in this investigation suggest that many putative viral sequences could correspond to novel viruses that are hosted by cotton. Future studies need to be performed to confirm molecular and biological properties of the dozens of putative viral sequences presented here. Strategies for these studies could include a more comprehensive sample collection in commercial or experimental fields, isolation and purification of known and novel viruses in cotton, and isolation of fungal organisms that may serve as hosts for some putative viruses presented in this analysis.

## Methods

### Sample collection

An illustration of the entire process, from data collection to sequence analyses, is presented in Supplementary Fig. 1. Cotton plant samples were collected in 2021 (except for Fairhope, Alabama, which was collected in 2022) from six states (Supplementary Fig. 2) in the southern United States (Alabama [Fairhope, Headland, Prattville, and Brewton], Florida [Jay and Quincy], Georgia [Tifton], Louisiana [Winnsboro], North Carolina [Jackson Springs], and South Carolina [Blackville]). The regions where samples were collected are located within the cotton belt of the United States and represent areas of high cotton production. These sites also correspond to university research stations participating in a collaborative effort to understand the epidemiology of CLRDV^14^. Leaves and petiole samples were collected from symptomatic and asymptomatic upland cotton previously cultivated at the aforementioned research stations (Supplementary Table 1) and subsequently sent for further analyses to the Auburn University Plant Diagnostics Lab in Auburn, AL. Samples were transported in Ziplock plastic bags placed inside an ice chest containing ice. Upon arrival, all samples were stored at −80 °C until further processing.

### RNA isolation and sequencing

For locations with multiple samples (Supplementary Table 1), pooled samples were prepared by combining approximately 0.5 g of tissue from each individual plant collected at that site. The combined tissues were homogenized into a fine powder under liquid nitrogen using a mortar and pestle. From this homogenized pool, a 100 mg representative subsample was aliquoted for total RNA extraction using the PureLink^®^ Plant RNA Reagent kit (Invitrogen™, cat. #12322-012, Waltham, MA). For locations with only a single sample (Quincy, Winnsboro, and Jackson Springs), no pooling was performed, and RNA was extracted directly from 100 mg of individual plant tissue. DNA was digested from total RNA using the on-column method described by the RNA Clean & Concentrator kit (Zymo Research, cat. #R1014, Irvine, CA). Concentration and quality were determined in a spectrophotometer (NanoDrop^®^) and Tape Station 4200 (Agilent Technologies, Santa Clara, CA), respectively. Samples containing a concentration ≥ 50 ng/µl and an RNA integrity number ≥ 6.5 were used to prepare 10 libraries. Subsamples from symptomatic and asymptomatic plants within the same location were pooled for RNA extraction and used for library preparation and sequencing (Supplementary Table 1). Ribosomal RNAs from both eukaryotes and prokaryotes were depleted from total RNA samples. The remaining RNAs were fragmented into 250–300 bp and reverse-transcribed into double-stranded cDNAs, which subsequently went through end repair, A-tailing, and ligation with Illumina adapters. After fragment size selection and PCR amplification, the metatranscriptome library was ready for library quality control and sequencing. Libraries were sequenced in a NovaSeq Illumina platform with pair-end 150 bp sequencing (PE150, Q30 ≥ 85% with an average of 15 Gb raw data per sample equivalent to > 100 million raw reads) (Table 1).

### Sequence quality assessment and assembly

The majority of bioinformatic analyses were performed using the Galaxy platform^30^. Data quality was assessed with ‘FastQC’^31^ after removing Illumina adapters with ‘Trimmomatic’ using the ‘paired-end’ reads from two separate files function, and Illuminaclip step^32^. Sequence reads were mapped to the host reference genome (Accession GCA_007990345.1, *Gossypium hirsutum*) using ‘Bowtie2’^33,34^. Non-host reads were extracted from the mapped reads by filtering unmapped reads using ‘Samtools View’^35^, and unmapped reads were converted to fastq format using ‘Samtools Fastx’^35^. The database of ‘Kraken’ tool was used to perform a preliminary identification of putative viral reads^36^(data not shown). After the Kraken report was generated, genome assembly of non-host reads was performed with ‘MetaSPAdes’^37^. To minimize the risk of chimeric assemblies, MetaSPAdes was used with its built-in algorithms for masking strain variation and disconnecting low-coverage artifacts. Obtained contigs were filtered with a threshold of > 400 nt long.

### Sequence analysis and clustering

To identify sequences of putative viruses, a discontiguous megablast (dc-megablast) analysis was performed using the ‘NCBI BLAST+ blastn’ software integrated into Galaxy^38,39^. A second iteration was performed using the ‘low complexity sequences’ algorithm in NCBI BLAST^®^^40^ and the NCBI Virus database^41,42^. The nucleotide completeness of partial and complete was determined by comparing their length to the highest-scoring reference viral sequence in the database. The taxonomic classification at the family level and host range was performed based on the NCBI virus database and confirmed with the most recent ICTV Taxonomy Report (https://ictv.global/report/about, as of July 2024). If available, information of the genus and species classification was assigned based on the NCBI Virus database and Blastn search. The percent identity and query coverage were determined, and contigs were classified into three groups based on the percent query coverage: 70–100%, 50–69%, and < 50% (Supplementary Tables 2–11). Contigs with no significant similarity value and hits marked as bacteriophage were excluded for further analyses.

### Sequence validation by RT-PCR

A total of 12 sequences were selected, including viruses of interest, putative viruses frequently detected in 9 of the 10 locations, and nearly complete genomes (Supplementary Table 13). Two sets of primers were designed for each selected sequence (Supplementary Tables 12 and 13) using the Primer-BLAST tool^43^. Following optimization via gradient PCR, the most effective primer set for each target was selected for the final validation (Supplementary Table 12). For CLRDV, RT-PCR was performed using a primer set previously published^44^. A two-step RT-PCR system was performed using the SuperScript IV First-Strand Synthesis System (Invitrogen™, cat. #18091050) for synthesis of first-strand cDNA from total plant RNA. The DNA polymerase reaction was performed using the Platinum™ Taq DNA Polymerase kit (Invitrogen™, cat. #10966018). PCR products were analyzed for size profiling in an automated electrophoresis platform (TapeStation 4200, Agilent Technologies) (Fig. 1D). Samples with the expected PCR product size were Sanger-sequenced, and the obtained sequences were analyzed and edited using Geneious Prime (version 2023.2.1).

### Phylogenetic analysis

Virus families with the highest abundance across locations (≥ 0.8, Fig. 3) were initially selected for phylogenetic analysis. However, given the mitochondrial genetic code of Mitoviruses^45^, sequences of this family were excluded, and analyses were conducted only on *Botourmiaviridae*, *Partitiviridae*, and *Solemoviridae* viruses. Because all *Solemoviridae* viruses identified in this study were of the same species (CLRDV), whole genome sequences were used, which were generally more divergent than the RdRp coding regions, to allow better separation of different CLRDV isolates phylogenetically. For the *Botourmiaviridae* and *Partitiviridae* sequences, Blastn searches indicated each family was comprised of multiple species, hence their deduced RdRp amino acid sequences with minimum length of 400 aa predicted in each location (if all sequences were shorter than 400-aa in a location, only the longest one was selected) were retrieved to construct phylogenetic trees. Sequences (i.e. whole genomes for CLRDVs and RdRp amino acids for *Botourmiaviridae* and *Partitiviridae*) were first aligned using MAFFT version 7.505^46^ with default settings. Poorly aligned regions were trimmed using TRIMAL version 1.2^47^ based on a gap threshold of 0.10 for CLRDVs and 0.25 for *Botourmiaviridae* and *Partitiviridae* viruses. The evolutionary model-of-best-fit for CLRDVs, GTR + I+G, was determined according to the Bayesian Information Criterion (BIC) on MEGA11^48^. For *Botourmiaviridae* and *Partitiviridae*, mixed protein models were adopted. Phylogenetic analyses were run on MRBAYES (version 3.2.7). Two runs with four chains per run were executed until the standard deviation of split frequencies between runs dropped below 0.05. The first 25% of generations were discarded, and the remaining generations were used to build 50% majority-rule consensus trees, which were visualized on FigTree software (version 1.4.4).

## Supplementary Information

Below is the link to the electronic supplementary material.

Supplementary Material 1

## Data Availability

The datasets generated during and/or analyzed during the current study are available at the SRA BioProject accession number PRJNA1015735 (https://www.ncbi.nlm.nih.gov/sra/PRJNA1015735). The complete putative viral sequences in FASTA format and the Sanger sequences from PCR products are publicly available in the GitHub repository (https://github.com/ceesgua91/Cotton_virome.git).
