# Supplementary material for: Metatranscriptomics analysis reveals the cotton virome in the southern United States
Source: Sci Rep. 2026 Feb 23;16:10229. doi: 10.1038/s41598-026-40828-5 (PMC13031293; doi:10.1038/s41598-026-40828-5)
Supplement: Supplementary file 1 — Supplementary Material 1 [file 41598_2026_40828_MOESM1_ESM.docx]

| **Sample No.** | **State/Location** | **Symptomatic/Asymptomatic** | **Collection date** | **Sampling period** |
| --- | --- | --- | --- | --- |
| 1 | Alabama-Brewton | Symptomatic | 6.29.21 | 60 DAE |
| 2 | Alabama-Brewton | Symptomatic | 7.27.21 | 90 DAE |
| 3 | Alabama-Brewton | Symptomatic | 7.27.21 | 90 DAE |
| 4 | Alabama-Brewton | Symptomatic | 8.23.21 | 120 DAE |
| 5 | Alabama-Brewton | Symptomatic | 8.23.21 | 120 DAE |
| 6 | Alabama-Brewton | Symptomatic | 7.27.21 | 60 DAE |
| 7 | Alabama-Brewton | Symptomatic | 8.23.21 | 90 DAE |
| 8 | Alabama-Brewton | Symptomatic | 9.22.21 | 120 DAE |
| 9 | Alabama-Brewton | Symptomatic | 9.22.21 | 120 DAE |
| 10 | Alabama-Prattville | Symptomatic | 7.28.21 | 90 DAE |
| 11 | Alabama-Prattville | Symptomatic | 7.28.21 | 90 DAE |
| 12 | Alabama-Prattville | Symptomatic | 7.28.21 | 90 DAE |
| 13 | Alabama-Prattville | Symptomatic | 7.28.21 | 90 DAE |
| 14 | Alabama-Prattville | Asymptomatic | 7.28.21 | 60 DAE |
| 15 | Alabama-Prattville | Asymptomatic | 7.28.21 | 60 DAE |
| 16 | Alabama-Prattville | Symptomatic | 7.28.21 | 60 DAE |
| 17 | Alabama-Prattville | Symptomatic | 7.28.21 | 60 DAE |
| 18 | Alabama-Prattville | Symptomatic | 8.23.21 | 90 DAE |
| 19 | Alabama-Prattville | Symptomatic | 8.23.21 | 90 DAE |
| 20 | Alabama-Headland | Symptomatic | 9.24.21 | 120 DAE |
| 21 | Alabama-Headland | Symptomatic | 8.3.22 | 60 DAE |
| 22 | Alabama-Headland | Asymptomatic | 9.3.21 | 90 DAE |
| 23 | Alabama-Headland | Symptomatic | 9.3.21 | 90 DAE |
| 24 | Alabama-Headland | Symptomatic | 9.3.21 | 90 DAE |
| 25 | Alabama-Headland | Asymptomatic | 9.3.21 | 90 DAE |
| 26 | Alabama-Headland | Symptomatic | 10.1.21 | 120 DAE |
| 27 | Alabama-Headland | Symptomatic | 10.1.21 | 120 DAE |
| 28 | Alabama-Fairhope | Symptomatic | 7.15.22 | ND |
| 29 | Alabama-Fairhope | Symptomatic | 7.15.22 | ND |
| 30 | Alabama-Fairhope | Symptomatic | 7.15.22 | ND |
| 31 | Alabama-Fairhope | Symptomatic | 7.15.22 | ND |
| 32 | Alabama-Fairhope | Symptomatic | 7.15.22 | ND |
| 33 | Florida-Jay | Asymptomatic | 10.1.21 | 90 DAE |
| 34 | Florida-Jay | Asymptomatic | 8.18.21 | 90 DAE |
| 35 | Florida-Jay | Asymptomatic | 8.18.21 | 120 DAE |
| 36 | Florida-Jay | Asymptomatic | 8.18.21 | 120 DAE |
| 37 | Florida-Quincy | ND | 10.4.21 | 90 DAE |
| 38 | Georgia-Tifton | Asymptomatic | 8.18.21 | 60 DAE |
| 39 | Georgia-Tifton | Asymptomatic | 10.5.21 | 120 DAE |
| 40 | Georgia-Tifton | Asymptomatic | 10.5.21 | 120 DAE |
| 41 | Louisiana-Winnsboro | Asymptomatic | 8.25.21 | 90 DAE |
| 42 | North Carolina-Jackson Springs | Symptomatic | 8.15.21 | 90 DAE |
| 43 | South Carolina- Blackville | ND | 8.16.21 | 90 DAE |
| 44 | South Carolina- Blackville | ND | 8.16.21 | 90 DAE |
| 45 | South Carolina- Blackville | ND | 8.16.21 | 90 DAE |
| 46 | South Carolina- Blackville | ND | 8.16.21 | 90 DAE |

*Continued from last previous page*. Supplementary Table 1. List of samples used in this study. Samples were pooled per location before performing RNA extraction and library preparation. DAE= days after seed emergence, ND= not determined.

| **Contig No.** | **Length (bp)** | **Genome**  **completeness** | **Putative virus name** | **Identity (%)** | **Query cover (%)** | **Type** | **Accession** | **Known hosts** | **Family** | **Genus** |
| --- | --- | --- | --- | --- | --- | --- | --- | --- | --- | --- |
| 22 | 3130 | Complete | Ustilaginoidea virens narna-like virus 1 | 82.02 | 100 | ssRNA | ON791652.1 | Fungi | Narnaviridae | No rank |
| 123 | 2140 | Partial | Bacteriophage sp. | 99.86 | 100 | DNA | MN855681.1 | Bacteria | Bacteriophage | No rank |
| 761 | 1337 | Partial | Caudoviricetes sp. | 99.93 | 100 | DNA | MN855675.1 | Bacteria | Bacteriophage | No rank |
| 873 | 1286 | Partial | Escherichia phage Lambda | 100 | 100 | DNA | OR974322.1 | Bacteria | Bacteriophage | Lambdavirus |
| 934 | 1262 | Partial | Bacteriophage sp. | 99.92 | 100 | DNA | BK024015.1 | Bacteria | Bacteriophage | No rank |
| 1198 | 1176 | Partial | Caudoviricetes sp. | 99.91 | 100 | DNA | BK035681.1 | Bacteria | Bacteriophage | No rank |
| 1366 | 1134 | Partial | Bacteriophage sp. | 100 | 100 | DNA | MN855678.1 | Bacteria | Bacteriophage | No rank |
| 1377 | 1132 | Partial | Bacteriophage sp. | 99.91 | 100 | DNA | BK024015.1 | Bacteria | Bacteriophage | No rank |
| 1447 | 1114 | Partial | Bacteriophage sp. | 100 | 100 | DNA | MN855678.1 | Bacteria | Bacteriophage | No rank |
| 1686 | 1063 | Partial | Bacteriophage sp. | 99.81 | 100 | DNA | MN855678.1 | Bacteria | Bacteriophage | No rank |
| 1740 | 1052 | Partial | Bacteriophage sp. | 100 | 100 | DNA | MN855681.1 | Bacteria | Bacteriophage | No rank |
| 13223 | 485 | Partial | Caudoviricetes sp. | 100 | 100 | DNA | MN855675.1 | Bacteria | Bacteriophage | No rank |
| 1 | 5842 | Complete | Cotton leafroll dwarf virus | 98.75 | 99 | ssRNA | OQ300130.1 | Plants | Solemoviridae | Polerovirus |
| 86 | 2366 | Complete | Botourmiaviridae sp. | 88.34 | 99 | ssRNA | ON812916.1 | Plants, Fungi | Botourmiaviridae | No rank |
| 94 | 2336 | Complete | Botourmiaviridae sp. | 95.68 | 99 | ssRNA | ON812923.1 | Plants, Fungi | Botourmiaviridae | No rank |
| 328 | 1693 | Partial | Botourmiaviridae sp. | 96.22 | 99 | ssRNA | ON813040.1 | Plants, Fungi | Botourmiaviridae | No rank |
| 343 | 1677 | Partial | Botourmiaviridae sp. | 89.78 | 99 | ssRNA | ON813041.1 | Plants, Fungi | Botourmiaviridae | No rank |
| 100 | 2288 | Complete | Guiyang botourmia-like virus 3 | 93.39 | 97 | ssRNA | OM514661.1 | Plants, Fungi | Botourmiaviridae | No rank |
| 20 | 3148 | Complete | Narnaviridae sp. | 73.63 | 96 | ssRNA | ON812068.1 | Fungi | Narnaviridae | No rank |
| 345 | 1674 | Partial | Erysiphe necator associated partitivirus 7 | 76.47 | 96 | dsRNA | MN605490.1 | Plants, Fungi | Partitiviridae | No rank |
| 1320 | 1146 | Partial | Botourmiaviridae sp. | 73.13 | 96 | RNA | ON585090.1 | Fungi | Botourmiaviridae | No rank |
| 1027 | 1227 | Complete | Erysiphe necator associated narnavirus 31 | 75.95 | 95 | ssRNA | MN605444.1 | Fungi | Narnaviridae | Narnavirus |
| 263 | 1793 | Partial | XiangYun narna-levi-like virus 10 | 73.76 | 87 | RNA | OL700146.1 | Insects | unclassified Riboviria | No rank |
| 779 | 1326 | Partial | Colletotrichum gloeosporioides partitivirus 1 | 68.01 | 87 | dsRNA | MK926567.1 | Fungi | Partitiviridae | No rank |
| 95 | 2331 | Partial | Plasmopara viticola lesion associated polymycovirus 3 | 69.14 | 85 | dsRNA | MN557034.1 | Fungi | unclassified Riboviria | No rank |
| 73 | 2462 | Complete | Leptosphaeria biglobosa botourmiavirus 2 | 66.62 | 84 | ssRNA | OP441680.1 | Plants, Fungi | Botourmiaviridae | No rank |
| 5241 | 716 | Partial | Cauliflower mosaic virus | 100 | 84 | DNA | NC_001497.2 | Plants | Caulimoviridae | Caulimovirus |
| 80 | 2419 | Complete | Botourmiaviridae sp. | 69.25 | 80 | ssRNA | ON812915.1 | Plants, Fungi | Botourmiaviridae | No rank |
| 713 | 1362 | Partial | Caudoviricetes sp. | 100 | 78 | DNA | BK028718.1 | Bacteria | Bacteriophage | No rank |
| 2210 | 978 | Partial | Agaricus bisporus virus X | 96.28 | 74 | ssRNA | AJ421989.1 | Fungi | unclassified Riboviria | No rank |
| 27 | 3036 | Complete | Plant associated botourmia-like virus 1 | 85.35 | 68 | ssRNA | OL472278.1 | Plants | Botourmiaviridae | No rank |
| 8788 | 581 | Partial | Plasmopara viticola lesion associated Partitivirus 4 | 96.67 | 56 | dsRNA | MN556976.1 | Fungi | Partitiviridae | No rank |
| 1673 | 1066 | Partial | Bacteriophage sp. | 100 | 42 | DNA | BK024015.1 | Bacteria | Bacteriophage | No rank |
| 71 | 2497 | Partial | Cassava vein mosaic virus | 99.81 | 20 | dsDNA | NC_001648.1 | Plants | Caulimoviridae | Caulimovirus |
| 7 | 3886 | Complete | Leviviridae sp. | 100 | 2 | ssRNA | MZ679536.1 | Bacteria | Bacteriophage | No rank |
| 68 | 2506 | Partial | White spot syndrome virus | 74.44 | ND | dsDNA | MH883318.1 | Crustaceans | ND | ND |
| 1456 | 1113 | Partial | Caudoviricetes sp. | 100 | ND | DNA | BK035681.1 | Bacteria | ND | ND |

Supplementary Table 2. Putative viral sequence information for the Fairhope, Alabama library. Sequence data can be found in fasta format in Github under a file named “AL4_Alabama-Fairhope.” Red font indicates bacteriophage sequences. Red font with light red fill color are sequences with no significant similarity found with NCBI Blastn search. Green, blue, and gold fill colors are sequences with query coverage of 70-100%, 50-69%, and <50%, respectively. ND= not determined.

| **Contig No.** | **Length (bp)** | **Genome**  **completeness** | **Putative virus name** | **Identity (%)** | **Query cover (%)** | **Type** | **Accession** | **Known hosts** | **Family** | **Genus** |
| --- | --- | --- | --- | --- | --- | --- | --- | --- | --- | --- |
| 1704 | 1330 | Partial | Hulunbuir Botou tick virus 7 | 93.48 | 100 | ssRNA(+) | ON746357.1 | Invertebrates | Botourmiaviridae | No rank |
| 9851 | 751 | Partial | XiangYun narna-levi-like virus 10 | 79.23 | 100 | RNA | OL700146.1 | Insects | unclassified Riboviria | No rank |
| 25358 | 508 | Partial | Plasmopara viticola lesion associated Partitivirus 4 | 99.21 | 100 | dsRNA | MN556976.1 | Fungi | Partitiviridae | No rank |
| 4 | 5887 | Complete | Cotton leafroll dwarf virus | 99.52 | 99 | ssRNA(+) | OQ300127.1 | Plants | Solemoviridae | Polerovirus |
| 159 | 2433 | Partial | Diaporthe gulyae mitovirus 2 | 71.75 | 99 | ssRNA(+) | OR224967.1 | Fungi | Mitoviridae | Mitovirus |
| 2370 | 1207 | Partial | XiangYun narna-levi-like virus 10 | 77.28 | 99 | RNA | OL700146.1 | Insects | unclassified Riboviria | No rank |
| 21692 | 544 | Partial | Botourmiaviridae sp. | 75.54 | 99 | ssRNA(+) | ON813052.1 | Invertebrates | Botourmiaviridae | No rank |
| 28768 | 482 | Partial | Helminthosporium victoriae virus 190S | 78.5 | 99 | dsRNA | NC_003607.2 | Fungi | Totiviridae | Victorivirus |
| 29347 | 478 | Partial | Alternaria dianthicola negative-stranded RNA virus 1 | 79.62 | 99 | RNA | ON843764.1 | Fungi | unclassified Riboviria | No rank |
| 31923 | 461 | Partial | Grapevine partitivirus | 92.86 | 99 | dsRNA | JX658570.1 | Plants | Partitiviridae | No rank |
| 38090 | 428 | Partial | Bipolaris maydis victorivirus 1b | 75.76 | 99 | dsRNA | MN935503.1 | Fungi | Totiviridae | Victorivirus |
| 42094 | 409 | Partial | Erysiphe necator associated ourmia-like virus 56 | 72.82 | 99 | ssRNA(+) | MN611584.1 | Fungi | Botourmiaviridae | Ourmiavirus |
| 43629 | 403 | Partial | Delitschia confertaspora partitivirus 1 | 79.6 | 99 | dsRNA | MK279447.1 | Fungi | Partitiviridae | No rank |
| 44221 | 400 | Partial | Alternaria arborescens mitovirus 1 | 91.98 | 99 | ssRNA(+) | ON714134.1 | Fungi | Mitoviridae | Unuamitovirus |
| 130 | 2552 | Complete | Erysiphe necator associated ourmia-like virus 69 | 95.15 | 98 | ssRNA(+) | ON811984.1 | Fungi | Botourmiaviridae | Ourmiavirus |
| 30692 | 469 | Partial | Botourmiaviridae sp. | 73.33 | 98 | ssRNA(+) | ON813052.1 | Invertebrates | Botourmiaviridae | No rank |
| 32667 | 456 | Partial | Grapevine partitivirus | 88.99 | 98 | dsRNA | JX658570.1 | Plants | Partitiviridae | No rank |
| 17773 | 591 | Partial | XiangYun narna-levi-like virus 10 | 75.66 | 96 | RNA | OL700146.1 | Insects | unclassified Riboviria | No rank |
| 42168 | 409 | Partial | uncultured totivirus | 93.33 | 95 | dsRNA | HE579556.1 | Fungi | Totiviridae | No rank |
| 138 | 2502 | Partial | Soybean leaf-associated mitovirus 2 | 85.92 | 90 | ssRNA(+) | KT598239.1 | Fungi | Mitoviridae | Mitovirus |
| 206 | 2261 | Complete | Erysiphe necator associated mitovirus 23 | 74.33 | 90 | ssRNA(+) | MN611669.1 | Fungi | Mitoviridae | Mitovirus |
| 144 | 2487 | Complete | Alternaria arborescens mitovirus 1 | 73.28 | 87 | ssRNA(+) | MN599397.1 | Fungi | Mitoviridae | Unuamitovirus |
| 6440 | 879 | Partial | Agaricus bisporus virus X | 96.39 | 81 | Unknown | AJ421989.1 | Fungi | unclassified Riboviria | No rank |
| 1 | 6499 | Complete | Cladosporium cladosporioides negative-stranded RNA virus 1 | 69.43 | 70 | ssRNA(-) | MK584856.1 | Fungi | unclassified Riboviria | No rank |
| 5550 | 924 | Partial | Alternaria alternata chrysovirus 1 | 85.68 | 56 | dsRNA | NC_040737.1 | Fungi | Chrysoviridae | Betachrysovirus |
| 42564 | 407 | Partial | Hulunbuir Botou tick virus 3 | 81.38 | 44 | ssRNA(+) | ON746352.1 | Invertebrates | Botourmiaviridae | No rank |
| 24356 | 517 | Partial | Rhizoctonia solani hypovirus 11 | 84.27 | 17 | RNA | OM984482.1 | Fungi | Hypoviridae | Hypovirus |
| 14647 | 641 | Partial | Emiliania huxleyi virus 156 | 78.57 | 14 | dsDNA | KF481687.1 | Eukaryotic algae | Phycodnaviridae | Coccolithovirus |
| 32772 | 456 | Partial | Prochlorococcus phage P-TIM68 | 80.88 | 14 | DNA | KM359505.1 | Bacteria | Bacteriophage | Haifavirus |
| 41452 | 412 | Partial | Human alphaherpesvirus 2 | 91.49 | 11 | dsDNA | MF510315.1 | Vertebrates | Orthoherpesviridae | Simplexvirus |
| 24600 | 515 | Partial | Mollivirus sibericum | 94.44 | 6 | Unknown | KR921745.1 | Protozoa | unclassified Riboviria | No rank |
| 36119 | 438 | Partial | Muromegalovirus G4 | 86.27 | ND | dsDNA | MT886700.1 | Vertebrates | ND | ND |
| 41689 | 411 | Partial | Ectocarpus siliculosus virus 1 | 74.26 | ND | dsDNA | NC_002687.1 | Eukaryotic algae | ND | ND |

Supplementary Table 3. Putative viral sequence information for the Headland, Alabama library. Sequence data can be found in fasta format in Github under a file named “AL6_Alabama-Headland.” Red font indicates bacteriophage sequences. Red font with light red fill color are sequences with no significant similarity found with NCBI Blastn search. Green, blue, and gold fill colors are sequences with query coverage of 70-100%, 50-69%, and <50%, respectively. ND= not determined.

| **Contig No.** | **Length (bp)** | **Genome**  **completeness** | **Putative virus name** | **Identity (%)** | **Query cover (%)** | **Type** | **Accession** | **Known hosts** | **Family** | **Genus** |
| --- | --- | --- | --- | --- | --- | --- | --- | --- | --- | --- |
| 5025 | 951 | Partial | Potato yellow dwarf nucleorhabdovirus | 88.55 | 100 | ssRNA(-) | NC_076163.1 | Plants | Rhabdoviridae | Alphanucleorhabdovirus |
| 8045 | 791 | Partial | Potato yellow dwarf nucleorhabdovirus | 91.4 | 100 | ssRNA(-) | NC_076163.1 | Plants | Rhabdoviridae | Alphanucleorhabdovirus |
| 10689 | 702 | Partial | Diaporthe gulyae mitovirus 2 | 88.89 | 100 | ssRNA(+) | OR224967.1 | Fungi | Mitoviridae | Mitovirus |
| 21045 | 521 | Partial | Constricta yellow dwarf virus | 97.89 | 100 | ssRNA(-) | OP998261.1 | Plants | Rhabdoviridae | Alphanucleorhabdovirus |
| 21990 | 510 | Partial | Soybean leaf-associated mitovirus 3 | 98.43 | 100 | ssRNA(+) | KT598240.1 | Plants | Mitoviridae | Mitovirus |
| 22109 | 509 | Partial | Potato yellow dwarf nucleorhabdovirus | 91.75 | 100 | ssRNA(-) | NC_076163.1 | Plants | Rhabdoviridae | Alphanucleorhabdovirus |
| 28756 | 452 | Partial | Soybean leaf-associated mitovirus 3 | 97.35 | 100 | ssRNA(+) | KT598240.1 | Plants | Mitoviridae | Mitovirus |
| 33525 | 421 | Partial | Escherichia phage ZL19 | 99.76 | 100 | dsDNA | OM258170.1 | Bacteria | Drexlerviridae | No rank |
| 7 | 5974 | Complete | Cotton leafroll dwarf virus | 98.98 | 99 | ssRNA | MN071395.1 | Plants | Solemoviridae | Polerovirus |
| 805 | 1726 | Partial | Gammapartitivirus sp. | 89.19 | 99 | dsRNA | MZ556342.1 | Plants, fungi | Partitiviridae | Gammapartitivirus |
| 1112 | 1568 | Partial | Plasmopara viticola lesion associated partitivirus 3 | 84.94 | 99 | dsRNA | MN556982.1 | Fungi | Partitiviridae | No rank |
| 5692 | 907 | Partial | Potato yellow dwarf nucleorhabdovirus | 91.71 | 99 | ssRNA(-) | NC_076163.1 | Plants | Rhabdoviridae | Alphanucleorhabdovirus |
| 13657 | 632 | Partial | Potato yellow dwarf nucleorhabdovirus | 89.86 | 99 | ssRNA(-) | NC_076163.1 | Plants | Rhabdoviridae | Alphanucleorhabdovirus |
| 14188 | 621 | Partial | Potato yellow dwarf nucleorhabdovirus | 91.75 | 99 | ssRNA(-) | NC_076163.1 | Plants | Rhabdoviridae | Alphanucleorhabdovirus |
| 21824 | 512 | Partial | Agaricus bisporus virus X | 95.9 | 99 | Unknown | AJ421989.1 | Fungi | unclassified Riboviria | No rank |
| 26687 | 467 | Partial | Potato yellow dwarf nucleorhabdovirus | 90.56 | 99 | ssRNA(-) | NC_076163.1 | Plants | Rhabdoviridae | Alphanucleorhabdovirus |
| 28526 | 453 | Partial | Diaporthe gulyae mitovirus 2 | 87.86 | 99 | ssRNA(+) | OR224967.1 | Fungi | Mitoviridae | Mitovirus |
| 37361 | 400 | Partial | Potato yellow dwarf nucleorhabdovirus | 91.44 | 99 | ssRNA(-) | KY564176.1 | Plants | Rhabdoviridae | Alphanucleorhabdovirus |
| 34298 | 416 | Partial | Erysiphe necator associated mycovirus 2 | 88.05 | 98 | RNA | MN617790.1 | Fungi | unclassified Riboviria | No rank |
| 35515 | 410 | Partial | Diaporthe gulyae mitovirus 2 | 87.81 | 98 | ssRNA(+) | OR224967.1 | Fungi | Mitoviridae | Mitovirus |
| 405 | 2030 | Complete | Erysiphe necator associated narnavirus 11 | 83.66 | 97 | ssRNA(+) | MN605424.1 | Fungi | Narnaviridae | Narnavirus |
| 12214 | 662 | Partial | Soybean leaf-associated mitovirus 2 | 79.62 | 96 | ssRNA(+) | KT598239.1 | Plants | Mitoviridae | Mitovirus |
| 36950 | 403 | Partial | Alternaria brassicicola mitovirus | 77.46 | 95 | ssRNA(+) | NC_076575.1 | Fungi | Mitoviridae | Unuamitovirus |
| 3942 | 1040 | Partial | Diaporthe helianthi mitovirus 1 | 65.98 | 89 | ssRNA(+) | OR224984.1 | Fungi | Mitoviridae | Mitovirus |
| 846 | 1698 | Partial | Soybean leaf-associated mitovirus 2 | 89.27 | 88 | ssRNA(+) | KT598239.1 | Plants | Mitoviridae | Mitovirus |
| 1843 | 1339 | Partial | Phomopsis viticola mitovirus 961 | 67.26 | 83 | ssRNA(+) | MZ074321.1 | Fungi | Mitoviridae | Mitovirus |
| 10972 | 695 | Partial | Suillus luteus mitovirus 1 | 72.54 | 81 | ssRNA(+) | OQ862545.1 | Fungi | Mitoviridae | Mitovirus |
| 3174 | 1121 | Partial | Phomopsis viticola mitovirus 961 | 73.93 | 73 | ssRNA(+) | MZ074321.1 | Fungi | Mitoviridae | Mitovirus |
| 9661 | 732 | Partial | Botourmiaviridae sp. | 74.87 | 52 | ssRNA(+) | MZ679429.1 | Arthropods | Botourmiaviridae | No rank |
| 16403 | 581 | Partial | Emiliania huxleyi virus 86 | 79.22 | 13 | dsDNA | NC_007346.1 | Eukaryotic algae | ND | ND |
| 1822 | 1343 | Partial | Paramecium bursaria Chlorella virus CVR-1 | 81.97 | 4 | dsDNA | JX997164.1 | Eukaryotic algae | ND | ND |
| 33320 | 422 | Partial | Murid betaherpesvirus 1 | 99.76 | ND | dsDNA | BK063394.1 | Vertebrates | ND | ND |

Supplementary Table 4. Putative viral sequence information for the Prattville, Alabama library. Sequence data can be found in fasta format in Github under a file named “AL7_Alabama-Prattville.” Red font with light red fill color are sequences with no significant similarity found with NCBI Blastn search. Green and blue fill colors are sequences with query coverage of 70-100% and 50-69%, respectively. ND= not determined.

| **Contig No.** | **Length (bp)** | **Genome**  **completeness** | **Putative virus name** | **Identity (%)** | **Query cover (%)** | **Type** | **Accession** | **Known hosts** | **Family** | **Genus** |
| --- | --- | --- | --- | --- | --- | --- | --- | --- | --- | --- |
| 38 | 5303 | Complete | Erysiphe necator associated negative-stranded RNA virus 4 | 75.69 | 100 | RNA | ON812125.1 | Fungi, arthropods | Unclassified Riboviria | No rank |
| 1720 | 1989 | Complete | Neofusicoccum parvum narnavirus 2 | 81.73 | 100 | ssRNA(+) | MK584834.1 | Fungi | Narnaviridae | Narnavirus |
| 20934 | 767 | Partial | Plasmopara viticola lesion associated Partitivirus 4 | 95.83 | 100 | dsRNA | MN556976.1 | Fungi | Partitiviridae | No rank |
| 32336 | 617 | Partial | Grapevine partitivirus | 94 | 100 | dsRNA | JX658570.1 | Plants | Partitiviridae | No rank |
| 49089 | 497 | Partial | uncultured partitivirus | 95.58 | 100 | dsRNA | HE579549.1 | Fungi, plants | Partitiviridae | No rank |
| 52 | 4997 | Complete | Cotton leafroll dwarf virus | 99.52 | 99 | ssRNA | OQ300127.1 | Plants | Solemoviridae | Polerovirus |
| 9731 | 1087 | Partial | Erysiphe necator associated partitivirus 10 | 83.2 | 99 | dsRNA | MN605491.1 | Fungi | Partitiviridae | No rank |
| 13367 | 949 | Partial | Plasmopara viticola lesion associated polymycovirus 3 | 69.69 | 99 | RNA | MN557034.1 | Fungi | Unclassified Riboviria | No rank |
| 14308 | 919 | Partial | Erysiphe necator associated partitivirus 3 | 89.2 | 99 | dsRNA | ON812106.1 | Fungi | Partitiviridae | No rank |
| 15392 | 888 | Partial | Grapevine partitivirus | 89.24 | 99 | dsRNA | JX658570.1 | Plants | Partitiviridae | No rank |
| 21164 | 763 | Partial | Yichang Insect virus | 75.23 | 99 | ssRNA(-) | NC_031321.1 | Invertebrates, insects | Phenuiviridae | Goukovirus |
| 22155 | 746 | Partial | Cladosporium cladosporioides partitivirus 1 | 90.04 | 99 | dsDNA | OP946879.1 | Fungi | Partitiviridae | No rank |
| 24208 | 714 | Partial | Cotton leafroll dwarf virus | 98.87 | 99 | ssRNA(+) | OQ300123.1 | Plants | Solemoviridae | Polerovirus |
| 6558 | 1273 | Patial | Yichang Insect virus | 74.21 | 98 | ssRNA(-) | NC_031320.1 | Insects | Phenuiviridae | Goukovirus |
| 41990 | 539 | Partial | Erysiphe necator associated gammapartitivirus 2 | 80.38 | 98 | dsRNA | MN617788.1 | Fungi | Partitiviridae | Gammapartitivirus |
| 4068 | 1518 | Partial | XiangYun narna-levi-like virus 10 | 78.12 | 97 | RNA | OL700146.1 | Insects | Unclassified Riboviria | No rank |
| 22369 | 743 | Partial | Coniothyrium diplodiella negative-stranded RNA virus 1 | 79.03 | 97 | RNA | NC_078858.1 | Fungi | Discoviridae | Orthodiscovirus |
| 14 | 6406 | Partial | Zhangzhou tick virus 1 | 68.20 | 95 | ssRNA(-) | ON746482.1 | Fungi, arthropods | Peribunyaviridae | No rank |
| 42132 | 538 | Partial | Yichang Insect virus | 75.59 | 95 | ssRNA(-) | NC_031321.1 | Invertebrates, insects | Phenuiviridae | Goukovirus |
| 1034 | 2278 | Partial | Guiyang botourmia-like virus 2 | 89.82 | 94 | ssRNA(+) | OM514660.1 | Plants | Botourmiaviridae | No rank |
| 19606 | 791 | Partial | Yichang Insect virus | 69.73 | 87 | ssRNA(-) | NC_031322.1 | Invertebrates, insects | Phenuiviridae | Goukovirus |
| 41692 | 541 | Partial | Grapevine-associated botybirnavirus 1 | 74.53 | 87 | dsRNA | MW648438.1 | Plants | unclassified Riboviria | Botybirnavirus |
| 33995 | 602 | Partial | Leviviridae sp. | 66.48 | 86 | ssRNA(+) | ON162658.1 | Bacteria | Bacteriophage | No rank |
| 566 | 2672 | Complete | Botourmiaviridae sp. | 66.7 | 77 | ssRNA(+) | ON812867.1 | Arthropods | Botourmiaviridae | No rank |
| 33196 | 609 | Partial | Ipomoea aquatica botybirnavirus | 71.07 | 77 | dsRNA | MN841289.1 | Plants | unclassified Riboviria | Botybirnavirus |
| 29706 | 645 | Partial | Yunnan sediment hypovirus 2 | 69.3 | 68 | ssRNA(+) | MW896943.1 | Lake sediment | Hypoviridae | Hypovirus |
| 664 | 2566 | Complete | XiangYun narna-levi-like virus 10 | 68.08 | 66 | RNA | OL700146.1 | Insects | unclassified Riboviria | No rank |
| 17345 | 840 | Partial | Alternaria alternata polymycovirus 2 | 65.99 | 64 | RNA | OQ054004.1 | Fungi | Polymycoviridae | Polymycovirus |
| 33492 | 606 | Partial | Guiyang fiers-like virus 4 | 73.29 | 64 | ssRNA(+) | OM514498.1 | Bacteria | Bacteriophage | No rank |
| 27982 | 664 | Partial | Yichang Insect virus | 68.92 | 48 | ssRNA(-) | NC_031320.1 | Invertebrates, insects | Phenuiviridae | Goukovirus |
| 13592 | 942 | Partial | Leptosphaeria biglobosa botybirnavirus 1 | 68.26 | 46 | dsRNA | MZ612795.1 | Fungi | unclassified Riboviria | Botybirnavirus |
| 3524 | 1598 | Complete | Leviviridae sp. | 66.38 | 43 | ssRNA(+) | ON162494.1 | Bacteria | Bacteriophage | No rank |
| 33175 | 609 | Partial | Guiyang fiers-like virus 4 | 69.77 | 42 | ssRNA(+) | OM514498.1 | Bacteria | Bacteriophage | No rank |
| 177 | 3500 | Partial | Totiviridae sp. | 67.38 | 40 | dsRNA | MN033371.1 | Vertebrates | Totiviridae | No rank |
| 19962 | 785 | Partial | Erysiphe necator associated mitovirus 38 | 68.32 | 32 | ssRNA(+) | ON605608.1 | Fungi | Mitoviridae | Mitovirus |
| 169 | 3536 | Partial | Grapevine-associated levi-like virus 10 | 66.13 | 29 | ssRNA(+) | MW648446.1 | Bacteria | Bacteriophage | No rank |
| 35076 | 592 | Partial | Suid alphaherpesvirus 1 | 72.66 | 21 | dsDNA | OP589231.1 | Vertebrates | Orthoherpesviridae | Varicellovirus |
| 8344 | 1158 | Partial | Grapevine-associated botybirnavirus 1 | 72.77 | 18 | dsRNA | MW648437.1 | Fungi | Unclassified Riboviria | Botybirnavirus |
| 69901 | 412 | Partial | Human betaherpesvirus 6 | 86.21 | 14 | dsDNA | KY315555.2 | Vertebrates | Orthoherpesviridae | Roseolovirus |
| 40224 | 551 | Partial | Chinese giant salamander iridovirus | 79.73 | 13 | dsDNA | KF512820.1 | Vertebrates | Iridoviridae | Ranavirus |
| 23421 | 726 | Partial | Rhizoctonia solani hypovirus 11 | 83.15 | 12 | RNA | OM984482.1 | Fungi | Hypoviridae | Hypovirus |
| 66053 | 425 | Partial | Ruegeria phage vB_RpoS-V16 | 85.19 | 12 | dsDNA | NC_052969.1 | Bacteria | Bacteriophage | No rank |
| 12227 | 985 | Partial | Saimiriine gammaherpesvirus 2 | 81.36 | 5 | DNA? | M31964.1 | Vertebrates | Orthoherpesviridae | Rhadinovirus |
| 11405 | 1015 | Partial | Pandoravirus salinus | 91.3 | 4 | dsDNA | NC_022098.1 | Protozoa | Unclassified Riboviria | Pandoravirus |
| 16553 | 859 | Partial | Python nidovirus | 94.59 | 4 | ssRNA(+) | KJ935003.1 | Vertebrates | Unclassified Riboviria | No rank |
| 2888 | 1713 | Partial | Paramecium bursaria Chlorella virus CVB-1 | 92.86 | 2 | dsDNA | JX997160.1 | Ciliate | Phycodnaviridae | Chlorovirus |
| 37906 | 569 | Partial | Klosneuvirus KNV1 | 76.99 | ND | dsDNA | KY684109.1 | Environment | ND | ND |
| 21025 | 765 | Partial | Bovine alphaherpesvirus 5 | 77.33 | ND | dsDNA | MZ420492.1 | Vertebrates | ND | ND |
| 20650 | 773 | Partial | Yichang Insect virus | 65.34 | ND | ssRNA(-) | NC_031321.1 | Invertebrates, insects | ND | ND |
| 41686 | 541 | Partial | Acanthamoeba castellanii mimivirus | 84.31 | ND | dsDNA | AP017645.1 | Protozoa | ND | ND |
| 61564 | 441 | Partial | Emiliania huxleyi virus 86 | 70.39 | ND | dsDNA | NC_007346.1 | Eukaryotic algae | ND | ND |

*Continued from previous page*. Supplementary Table 5. Putative viral sequence information for the Brewton, Alabama library. Sequence data can be found in fasta format in Github under a file named “AL8_Alabama-Brewton.” Red font indicates bacteriophage sequences. Red font with light red fill color are sequences with no significant similarity found with NCBI Blastn search. Green, blue, and gold fill colors are sequences with query coverage of 70-100%, 50-69%, and <50%, respectively. ND= not determined.

| **Contig No.** | **Length (bp)** | **Genome**  **completeness** | **Putative virus name** | **Identity (%)** | **Query cover (%)** | **Type** | **Accession** | **Known hosts** | **Family** | **Genus** |
| --- | --- | --- | --- | --- | --- | --- | --- | --- | --- | --- |
| 2336 | 1225 | Partial | Peanut mottle virus | 98.53 | 100 | ssRNA(+) | KY350138.1 | Plants | Potyviridae | Potyvirus |
| 3310 | 1081 | Partial | Peanut mottle virus | 99.26 | 100 | ssRNA(+) | KY350138.1 | Plants | Potyviridae | Potyvirus |
| 6092 | 847 | Partial | Peanut mottle virus | 98.85 | 100 | ssRNA(+) | MT675966.1 | Plants | Potyviridae | Potyvirus |
| 6406 | 829 | Partial | Peanut mottle virus | 99.16 | 100 | ssRNA(+) | KY350138.1 | Plants | Potyviridae | Potyvirus |
| 21063 | 486 | Partial | Peanut mottle virus | 99.59 | 100 | ssRNA(+) | KY350138.1 | Plants | Potyviridae | Potyvirus |
| 2911 | 1136 | Partial | XiangYun narna-levi-like virus 10 | 77.61 | 99 | RNA | OL700146.1 | Insects | Unclassified Riboviria | No rank |
| 7002 | 798 | Partial | Botourmiaviridae sp. | 81.43 | 99 | ssRNA(+) | ON812867.1 | Arthropods | Botourmiaviridae | No rank |
| 25111 | 447 | Partial | Agaricus bisporus virus X | 76.06 | 99 | ssRNA(+) | AJ421987.1 | Fungi | Unclassified Riboviria | No rank |
| 18 | 5946 | Complete | Cotton leafroll dwarf virus | 99.11 | 98 | ssRNA | OQ300128.1 | Plants | Solemoviridae | Polerovirus |
| 3427 | 1068 | Partial | Peanut mottle virus | 99.81 | 97 | ssRNA(+) | KY350138.1 | Plants | Potyviridae | Potyvirus |
| 7667 | 768 | Partial | Peanut mottle virus | 98.26 | 97 | ssRNA(+) | KY350138.1 | Plants | Potyviridae | Potyvirus |
| 1650 | 1380 | Partial | Guiyang botourmia-like virus 2 | 87.37 | 95 | ssRNA(+) | OM514660.1 | Plants | Botourmiaviridae | No rank |
| 25543 | 443 | Partial | Pseudomonas phage H66 | 71.22 | 91 | dsDNA | NC_042342.1 | Bacteria | Bacteriophage | Hollowayvirus |
| 360 | 2290 | Complete | Erysiphe necator associated mitovirus 23 | 74.17 | 89 | ssRNA(+) | MN611669.1 | Fungi | Mitoviridae | Mitovirus |
| 10672 | 663 | Partial | Figwort mosaic virus | 99.52 | 80 | dsDNA-RT | NC_003554.1 | Plants | Caulimoviridae | Caulimovirus |
| 21747 | 478 | Partial | Streptomyces phage Watermoore | 70.42 | 74 | dsDNA | OR553908.1 | Bacteria | Bacteriophage | Samistivirus |
| 147 | 3123 | Complete | Leviviridae sp. | 68.19 | 39 | ssRNA | ON162753.1 | Bacteria | Bacteriophage | No rank |
| 30877 | 404 | Partial | Human gammaherpesvirus 4 | 75.68 | 27 | dsDNA | MH590466.1 | Vertebrates | Orthoherpesviridae | Lymphocryptovirus |
| 17468 | 532 | Partial | Leviviridae sp. | 75.41 | 22 | ssRNA | MN035272.1 | Bacteria | Bacteriophage | No rank |
| 13123 | 608 | Partial | Rhizoctonia solani hypovirus 11 | 84.27 | 14 | RNA | OM984482.1 | Fungi | Hypoviridae | Hypovirus |
| 15210 | 568 | Partial | Cafeteria roenbergensis virus BV-PW1 | 68.93 | ND | dsDNA | NC_014637.1 | Protozoa | ND | ND |
| 18843 | 513 | Partial | Human gammaherpesvirus 8 | 81.08 | ND | dsDNA | KT271467.1 | Vertebrates | ND | ND |
| 22362 | 472 | Partial | Human alphaherpesvirus 1 | 73.53 | ND | dsDNA | ON960057.1 | Vertebrates | ND | ND |

Supplementary Table 6. Putative viral sequence information for the Jay, Florida library. Sequence data can be found in fasta format in Github under a file named “FL9_Florida-Jay.” Red font indicates bacteriophage sequences. Red font with light red fill color are sequences with no significant similarity found with NCBI Blastn search. Green and gold fill colors are sequences with query coverage of 70-100%, and <50%, respectively. ND= not determined.

| **Contig No.** | **Length (bp)** | **Genome**  **completeness** | **Putative virus name** | **Identity (%)** | **Query cover (%)** | **Type** | **Accession** | **Known hosts** | **Family** | **Genus** |
| --- | --- | --- | --- | --- | --- | --- | --- | --- | --- | --- |
| 63679 | 746 | Partial | Sclerotinia sclerotiorum mitovirus 36 | 97.45 | 100 | ssRNA(+) | MT646380.1 | Fungi | Mitoviridae | Mitovirus |
| 65516 | 732 | Partial | Human alphaherpesvirus 1 | 100 | 100 | dsDNA | OR405519.1 | Vertebrates | Orthoherpesviridae | Simplexvirus |
| 143368 | 435 | Partial | Sclerotinia sclerotiorum mitovirus 36 | 97.24 | 100 | ssRNA(+) | MT646380.1 | Fungi | Mitoviridae | Mitovirus |
| 148547 | 425 | Partial | Agaricus bisporus virus X | 96.95 | 100 | unknown | AJ421989.1 | Fungi | Unclassified Riboviria | No rank |
| 311 | 5898 | Complete | Cotton leafroll dwarf virus | 99.32 | 99 | ssRNA(+) | OQ300123.1 | Plants | Solemoviridae | Polerovirus |
| 1819 | 3599 | Complete | Erysiphe necator associated umbra-like virus 4 | 92.21 | 99 | ssRNA(+) | MN627470.1 | Fungi | Tombusviridae | Umbravirus |
| 5912 | 2395 | Partial | Botourmiaviridae sp. | 91.03 | 99 | ssRNA(+) | ON812912.1 | Arthropods | Botourmiaviridae | No rank |
| 11187 | 1871 | Complete | Neofusicoccum parvum narnavirus 2 | 93.47 | 99 | ssRNA(+) | MK584834.1 | Fungi | Narnaviridae | Narnavirus |
| 16636 | 1572 | Partial | Plasmopara viticola lesion associated Partitivirus 3 | 92.25 | 99 | dsRNA | MN556982.1 | Fungi | Partitiviridae | No rank |
| 17126 | 1552 | Partial | Grapevine partitivirus | 91.3 | 99 | dsRNA | JX658570.1 | Plants | Partitiviridae | No rank |
| 11019 | 1882 | Complete | Neofusicoccum parvum narnavirus 2 | 93.08 | 98 | ssRNA(+) | MK584834.1 | Fungi | Narnaviridae | Narnavirus |
| 13058 | 1754 | Complete | Erysiphe necator associated partitivirus 3 | 94.9 | 98 | dsRNA | ON812106.1 | Fungi | Partitiviridae | No rank |
| 157117 | 409 | Partial | Plasmopara viticola lesion associated Partitivirus 4 | 96.23 | 97 | dsRNA | MN556976.1 | Fungi | Partitiviridae | No rank |
| 6300 | 2339 | Partial | Plasmopara viticola lesion associated polymycovirus 3 | 68.32 | 96 | RNA | MN557034.1 | Fungi | unclassified Riboviria | No rank |
| 160939 | 403 | Partial | Barley aphid RNA virus 2 | 81.07 | 93 | RNA | LC516836.1 | Insects | unclassified Riboviria | No rank |
| 5222 | 2511 | Partial | Soybean leaf-associated mitovirus 2 | 85.88 | 90 | ssRNA(+) | KT598239.1 | Plants | Mitoviridae | Mitovirus |
| 699 | 4752 | Partial | Tonghua Totiv tick virus 1 | 69.23 | 82 | dsRNA | ON746545.1 | Arthropods | Totiviridae | No rank |
| 15486 | 1625 | Partial | Botourmiaviridae sp. | 68.7 | 82 | ssRNA(+) | MZ679446.1 | Arthropods | Botourmiaviridae | No rank |
| 2654 | 3185 | Partial | Narnavirus sp. | 67.49 | 79 | ssRNA(+) | MN035214.1 | Fungi | Narnaviridae | Narnavirus |
| 32941 | 1109 | Partial | Tonghua Totiv tick virus 1 | 69.21 | 73 | dsRNA | ON746545.1 | Arthropods | Totiviridae | No rank |
| 144068 | 433 | Partial | Figwort mosaic virus | 100 | 72 | dsDNA-RT | NC_003554.1 | Plants | Caulimoviridae | Caulimovirus |
| 160732 | 403 | Partial | Human gammaherpesvirus 4 | 68.27 | 62 | dsDNA | MH590569.1 | Vertebrates | Orthoherpesviridae | Lymphocryptovirus |
| 129490 | 465 | Partial | Diplodia scrobiculata RNA virus 1 | 77.89 | 61 | dsRNA | NC_013699.1 | Fungi | Unclassified Riboviria | No rank |
| 106266 | 531 | Partial | Diplodia scrobiculata RNA virus 1 | 75.44 | 53 | dsRNA | NC_013699.1 | Fungi | Unclassified Riboviria | No rank |
| 47399 | 899 | Partial | Plasmopara viticola lesion associated ourmia-like virus 13 | 71.28 | 52 | ssRNA(+) | NC_076748.1 | Fungi | Botourmiaviridae | Magoulivirus |
| 1925 | 3533 | Partial | Totiviridae sp. | 67.46 | 39 | dsRNA | MN033371.1 | Vertebrates | Totiviridae | No rank |
| 158267 | 408 | Partial | Human gammaherpesvirus 8 | 79.43 | 36 | dsDNA | JQ619843.1 | Vertebrates | Orthoherpesviridae | Rhadinovirus |
| 7559 | 2183 | Partial | Plasmopara viticola lesion associated mitovirus 22 | 63.98 | 28 | RNA | MN539783.1 | Fungi | Mitoviridae | Mitovirus |
| 45725 | 919 | Partial | Aureococcus anophagefferens virus | 74.87 | 21 | dsDNA | OM876856.1 | Vertebrates | Schizomimiviridae | Kratosvirus |
| 103566 | 541 | Partial | Human DNA virus | 79.44 | 19 | DNA | NC_040309.1 | Vertebrates | Unclassified Riboviria | No rank |
| 49864 | 871 | Partial | Rosellinia necatrix megabirnavirus 1 | 79.03 | 14 | dsRNA | NC_013462.1 | Fungi | Megabirnaviridae | Megabirnavirus |
| 64458 | 740 | Partial | Emiliania huxleyi virus 86 | 73.33 | 13 | dsDNA | NC_007346.1 | Eukaryotic algae | Phycodnaviridae | Coccolithovirus |
| 68246 | 712 | Partial | Human gammaherpesvirus 4 | 75.26 | 13 | dsDNA | AP019062.1 | Vertebrates | Orthoherpesviridae | Lymphocryptovirus |
| 75400 | 667 | Partial | Rhizoctonia solani hypovirus 11 | 84.27 | 13 | RNA | OM984482.1 | Fungi | Hypoviridae | Hypovirus |
| 1587 | 3754 | Complete | Leviviridae sp. | 71.08 | 11 | ssRNA(+) | ON162494.1 | Bacteria | Bacteriophage | No rank |
| 131828 | 460 | Partial | Bacteriophage sp. | 87.5 | 10 | dsDNA | OP072948.1 | Bacteria | Bacteriophage | No rank |
| 125579 | 474 | Partial | Micromonas sp. | 88.89 | 9 | dsDNA | NC_014767.1 | Algae | Phycodnaviridae | Prasinovirus |
| 144828 | 432 | Partial | Paramecium bursaria Chlorella virus NE-JV-4 | 93.02 | 9 | dsDNA | JX997179.1 | Eukaryotic algae | Phycodnaviridae | Chlorovirus |
| 32675 | 1114 | Partial | Suid alphaherpesvirus 1 | 80 | 8 | dsDNA | KM189914.3 | Vertebrates | Orthoherpesviridae | Varicellovirus |
| 53938 | 830 | Partial | Bodo saltans virus | 84.29 | 8 | dsDNA | NC_075036.1 | Protozoa | Mimiviridae | Theiavirus |
| 99871 | 554 | Partial | Macacine alphaherpesvirus 1 | 86.96 | 8 | dsDNA | KY628983.1 | Vertebrates | Orthoherpesviridae | Simplexvirus |
| 14317 | 1685 | Partial | Emiliania huxleyi virus 164 | 75.44 | 7 | dsDNA | KF481688.1 | Eukaryotic algae | Phycodnaviridae | Coccolithovirus |
| 50265 | 867 | Partial | Paramecium bursaria Chlorella virus FR483 | 90.32 | 7 | dsDNA | NC_008603.1 | Eukaryotic algae | Phycodnaviridae | Chlorovirus |
| 133683 | 455 | Partial | Ateline alphaherpesvirus 1 | 97.22 | 7 | dsDNA | NC_034446.1 | Vertebrates | Orthoherpesviridae | Simplexvirus |
| 44874 | 929 | Partial | Paramecium bursaria Chlorella virus CVR-1 | 81.97 | 6 | dsDNA | JX997164.1 | Eukaryotic algae | Phycodnaviridae | Chlorovirus |
| 83215 | 626 | Partial | Serratia phage vB_SmaS_Bonzee | 92.68 | 6 | dsDNA | NC_074752.1 | Bacteria | Bacteriophage | No rank |
| 90884 | 590 | Partial | Human alphaherpesvirus 1 | 90.24 | 6 | dsDNA | LT594107.1 | Vertebrates | Orthoherpesviridae | Simplexvirus |
| 34604 | 1079 | Partial | Pandoravirus salinus | 86.27 | 4 | dsDNA | NC_022098.1 | Protozoa | Unclassified Riboviria | Pandoravirus |
| 31755 | 1132 | Partial | Macacine gammaherpesvirus 4 | 91.11 | 3 | dsDNA | NC_006146.1 | Vertebrates | Orthoherpesviridae | Lymphocryptovirus |
| 3893 | 2795 | Partial | Pandoravirus inopinatum | 83.93 | 2 | dsDNA | KP136319.1 | Protozoa | Unclassified Riboviria | Pandoravirus |
| 12775 | 1772 | Partial | Paramecium bursaria Chlorella virus CVB-1 | 92.86 | 2 | dsDNA | JX997160.1 | Eukaryotic algae | Phycodnaviridae | Chlorovirus |
| 9724 | 1982 | Partial | Suid alphaherpesvirus 1 | 94.87 | 1 | dsDNA | KU198433.1 | Vertebrates | Orthoherpesviridae | Varicellovirus |
| 21052 | 1401 | Partial | Ictalurid herpesvirus 1 | 84.62 | ND | dsDNA | NC_001493.2 | Vertebrates | ND | ND |
| 25181 | 1278 | Partial | Herpesvirus papio 2 | 71.57 | ND | dsDNA | KF908243.1 | Vertebrates | ND | ND |
| 34110 | 1088 | Partial | Human gammaherpesvirus 8 | 81.08 | ND | dsDNA | KT271467.1 | Vertebrates | ND | ND |
| 53985 | 830 | Partial | Anguillid herpesvirus 1 | 87.04 | ND | dsDNA | OM649903.1 | Vertebrates | ND | ND |
| 85559 | 614 | Partial | Caudoviricetes sp. | 77.3 | ND | dsDNA | MW202476.1 | Bacteria | ND | ND |
| 152049 | 418 | Partial | Klosneuvirus KNV1 | 77.05 | ND | dsDNA | KY684109.1 | Waste water | ND | ND |

*Continued from previous page*. Supplementary Table 7. Putative viral sequence information for the Quincy, Florida library. Sequence data can be found in fasta format in Github under a file named “FL10_Florida-Quincy.” Red font indicates bacteriophage sequences. Red font with light red fill color are sequences with no significant similarity found with NCBI Blastn search. Green, blue, and gold fill colors are sequences with query coverage of 70-100%, 50-69%, and <50%, respectively. ND= not determined.

| **Contig No.** | **Length (bp)** | **Genome**  **completeness** | **Putative virus name** | **Identity (%)** | **Query cover (%)** | **Type** | **Accession** | **Known hosts** | **Family** | **Genus** |
| --- | --- | --- | --- | --- | --- | --- | --- | --- | --- | --- |
| 14 | 5966 | Complete | Cotton leafroll dwarf virus | 98.93 | 100 | RNA | OK185946.1 | Plants | Solemoviridae | Polerovirus |
| 587 | 1633 | Partial | Soybean thrips virus 3 | 81.43 | 100 | RNA | MW033629.1 | Insects | unclassified Riboviria | No rank |
| 4510 | 775 | Partial | Grapevine partitivirus | 93.55 | 100 | dsRNA | JX658570.1 | Plants | Partitiviridae | No rank |
| 6521 | 666 | Partial | Wuhan insect virus 21 | 96.25 | 100 | RNA | MT240792.1 | Insects | unclassified Riboviria | No rank |
| 7798 | 616 | Partial | Wuhan insect virus 21 | 97.08 | 100 | RNA | MT240792.1 | Insects | unclassified Riboviria | No rank |
| 9092 | 575 | Partial | Human alphaherpesvirus 1 | 100 | 100 | dsDNA | OR405519.1 | Vertebrates | Orthoherpesviridae | Simplexvirus |
| 15041 | 454 | Partial | Mammarenavirus guanaritoense | 84.21 | 100 | ssRNA | KU746283.1 | Vertebrates | Arenaviridae | Mammarenavirus |
| 18397 | 410 | Partial | Erysiphe necator associated mitovirus 23 | 74.7 | 100 | ssRNA(+) | MN611669.1 | Arthropods | Mitoviridae | Mitovirus |
| 3 | 9738 | Complete | Aphid lethal paralysis virus | 92.95 | 99 | RNA | LN907586.1 | Insects | Dicistroviridae | Cripavirus |
| 3078 | 903 | Partial | Grapevine partitivirus | 89.39 | 99 | dsRNA | JX658570.1 | Plants | Partitiviridae | No rank |
| 8949 | 579 | Partial | Wuhan insect virus 21 | 93.41 | 99 | RNA | MT240792.1 | Insects | unclassified Riboviria | No rank |
| 14781 | 458 | Partial | Wuhan insect virus 21 | 92.75 | 99 | RNA | MT240792.1 | Insects | unclassified Riboviria | No rank |
| 15065 | 454 | Partial | Aphid lethal paralysis virus | 97.79 | 99 | RNA | MF535297.1 | Insects | Dicistroviridae | Cripavirus |
| 16512 | 434 | Partial | Wuhan insect virus 21 | 97.92 | 99 | RNA | LC516848.1 | Insects | unclassified Riboviria | No rank |
| 1115 | 1312 | Partial | Soybean thrips virus 5 | 89.55 | 94 | RNA | MW033632.1 | Insects | unclassified Riboviria | No rank |
| 993 | 1365 | Partial | Soybean thrips virus 4 | 76.31 | 93 | RNA | MW033626.1 | Insects | unclassified Riboviria | No rank |
| 216 | 2331 | Partial | Guiyang botourmia-like virus 2 | 88.94 | 92 | ssRNA(+) | OM514660.1 | Plants | Botourmiaviridae | No rank |

Supplementary Table 8. Putative viral sequence information for the Tifton, Georgia library. Sequence data can be found in fasta format in Github under a file named “GA5_Georgia-Tifton.” Green fill color are sequences with query coverage of 70-100%.

| **Contig No.** | **Length (bp)** | **Genome**  **completeness** | **Putative virus name** | **Identity (%)** | **Query cover (%)** | **Type** | **Accession** | **Known hosts** | **Family** | **Genus** |
| --- | --- | --- | --- | --- | --- | --- | --- | --- | --- | --- |
| 466 | 5853 | Complete | Cotton leafroll dwarf virus | 99.49 | 100 | ssRNA | OK185941.1 | Plants | Solemoviridae | Polerovirus |
| 1838 | 2854 | Complete | Erysiphe necator associated ourmia-like virus 55 | 96.5 | 100 | ssRNA(+) | MN611583.1 | Fungi | Botourmiaviridae | Ourmiavirus |
| 5013 | 1796 | Partial | Alternaria arborescens mitovirus 1 | 93.04 | 100 | ssRNA(+) | ON714134.1 | Fungi | Mitoviridae | Unuamitovirus |
| 5622 | 1701 | Partial | Coniothyrium minitans RNA virus | 93.83 | 100 | dsRNA | KT598230.1 | Fungi, plants | Totiviridae | Victorivirus |
| 7709 | 1447 | Partial | Erysiphe necator associated partitivirus 10 | 84.89 | 100 | dsRNA | MN605491.1 | Plants, fungi | Partitiviridae | No rank |
| 10749 | 1206 | Complete | Neofusicoccum parvum narnavirus 2 | 95.61 | 100 | ssRNA(+) | MK584834.1 | Fungi | Narnaviridae | Narnavirus |
| 11996 | 1135 | Partial | Coniothyrium minitans RNA virus | 89.43 | 100 | dsRNA | KT598230.1 | Plants, fungi | Totiviridae | Victorivirus |
| 13069 | 1084 | Partial | Alternaria alternata chrysovirus 1 | 93.73 | 100 | dsRNA | MK584808.1 | Fungi | Chrysoviridae | Betachrysovirus |
| 17507 | 916 | Partial | Neofusicoccum parvum narnavirus 2 | 94.21 | 100 | ssRNA(+) | MK584834.1 | Fungi | Narnaviridae | Narnavirus |
| 20579 | 833 | Partial | Coniothyrium minitans RNA virus | 97.12 | 100 | dsRNA | KT598230.1 | Plants, fungi | Totiviridae | Victorivirus |
| 21352 | 815 | Partial | Alternaria alternata chrysovirus 1 | 99.14 | 100 | dsRNA | NC_040737.1 | Fungi | Chrysoviridae | Betachrysovirus |
| 25429 | 734 | Partial | Alternaria arborescens mitovirus 1 | 89.73 | 100 | ssRNA(+) | MN599397.1 | Fungi | Mitoviridae | Unuamitovirus |
| 50025 | 492 | Partial | Soybean leaf-associated mitovirus 3 | 96.54 | 100 | ssRNA(+) | KT598240.1 | Fungi | Mitoviridae | Mitovirus |
| 55840 | 461 | Partial | Plasmopara viticola lesion associated narnavirus 44 | 95.01 | 100 | ssRNA(+) | OQ990760.1 | Fungi | Narnaviridae | Narnavirus |
| 55871 | 461 | Partial | Plasmopara viticola lesion associated narnavirus 44 | 95.01 | 100 | ssRNA(+) | OQ990760.1 | Fungi | Narnaviridae | Narnavirus |
| 68364 | 410 | Partial | Leptosphaeria biglobosa hypovirus 1 | 88.29 | 100 | RNA | OP441692.1 | Fungi | Hypoviridae | Hypovirus |
| 68376 | 410 | Partial | uncultured chrysovirus | 99.02 | 100 | dsRNA | HE579619.1 | Fungi, plants, insects | Chrysoviridae | Chrysovirus |
| 1547 | 3107 | Complete | Leptosphaeria biglobosa mitovirus 6 | 78.59 | 99 | RNA | OP441675.1 | Fungi | Mitoviridae | Mitovirus |
| 2460 | 2484 | Complete | Alternaria arborescens mitovirus 1 | 91.49 | 99 | ssRNA(+) | ON714134.1 | Fungi | Mitoviridae | Unuamitovirus |
| 2501 | 2465 | Partial | Coniothyrium minitans RNA virus | 91.76 | 99 | dsRNA | KT598230.1 | Fungi, plants | Totiviridae | Victorivirus |
| 5524 | 1715 | Partial | Coniothyrium minitans RNA virus | 93.06 | 99 | dsRNA | KT598230.1 | Fungi, plants | Totiviridae | Victorivirus |
| 12727 | 1100 | Partial | Erysiphe necator associated partitivirus 3 | 90.13 | 99 | dsRNA | ON812106.1 | Plants, fungi | Partitiviridae | No rank |
| 17928 | 905 | Partial | Beauveria bassiana dsRNA mycovirus 1 | 78.59 | 99 | dsRNA | ON938188.1 | Fungi | unclassified Riboviria | No rank |
| 27695 | 698 | Partial | Soybean leaf-associated mitovirus 2 | 80.51 | 99 | ssRNA(+) | KT598239.1 | Fungi | Mitoviridae | Mitovirus |
| 30020 | 665 | Partial | Plasmopara viticola lesion associated Partitivirus 10 | 89.29 | 99 | dsRNA | ON812107.1 | Plants, fungi | Partitiviridae | No rank |
| 38985 | 571 | Partial | Enterobacteria phage mEp235 | 78.13 | 99 | dsDNA | NC_019708.1 | Bacteria | Bacteriophage | Nochtlivirus |
| 62461 | 432 | Partial | Erysiphe necator associated partitivirus 10 | 91.4 | 99 | dsRNA | MN605491.1 | Plants, fungi | Partitiviridae | No rank |
| 62968 | 430 | Partial | Soybean leaf-associated mitovirus 3 | 97.67 | 99 | ssRNA(+) | KT598240.1 | Fungi | Mitoviridae | Mitovirus |
| 71641 | 400 | Partial | Leptosphaeria biglobosa botybirnavirus 1 | 69.83 | 99 | dsRNA | MZ612796.1 | Fungi | unclassified Riboviria | Botybirnavirus |
| 2417 | 2507 | Complete | Jinan Botou tick virus 1 | 84.48 | 98 | ssRNA(+) | ON746369.1 | Arthropods, plants, fungi | Botourmiaviridae | No rank |
| 5267 | 1756 | Partial | Soybean leaf-associated mitovirus 2 | 85.23 | 98 | ssRNA(+) | KT598239.1 | Fungi | Mitoviridae | Mitovirus |
| 13299 | 1074 | Partial | Beauveria bassiana dsRNA mycovirus 1 | 71.2 | 98 | dsRNA | ON938189.1 | Fungi | unclassified Riboviria | No rank |
| 24931 | 743 | Partial | Soybean leaf-associated mitovirus 2 | 77.31 | 98 | ssRNA(+) | KT598239.1 | Fungi | Mitoviridae | Mitovirus |
| 39868 | 563 | Partial | Leptosphaeria biglobosa hypovirus 1 | 87.7 | 98 | RNA | OP441692.1 | Fungi | Hypoviridae | Hypovirus |
| 7184 | 1503 | Partial | Guiyang botourmia-like virus 2 | 88.2 | 96 | ssRNA(+) | OM514660.1 | Fungi, plants | Botourmiaviridae | No rank |
| 3368 | 2144 | Complete | Erysiphe necator associated mitovirus 23 | 74.06 | 95 | ssRNA(+) | MN611669.1 | Fungi | Mitoviridae | Mitovirus |
| 2238 | 2608 | Partial | XiangYun narna-levi-like virus 10 | 74.81 | 90 | RNA | OL700146.1 | Insect | unclassified Riboviria | No rank |
| 2455 | 2486 | Complete | Alternaria arborescens mitovirus 1 | 73.28 | 87 | ssRNA(+) | MN599397.1 | Fungi | Mitoviridae | Unuamitovirus |
| 51269 | 485 | Partial | Erysiphe necator associated ourmia-like virus 97 | 67.46 | 84 | ssRNA(+) | OM514629.1 | Fungi | Botourmiaviridae | Ourmiavirus |
| 5738 | 1684 | Partial | Sanya botourmia-like virus 3 | 69.05 | 80 | ssRNA(+) | OM514616.1 | Fungi, plants | Botourmiaviridae | No rank |
| 24234 | 756 | Partial | Erysiphe necator associated ourmia-like virus 56 | 68.7 | 78 | ssRNA(+) | MN611584.1 | Fungi | Botourmiaviridae | Ourmiavirus |
| 16532 | 948 | Partial | Beauveria bassiana dsRNA mycovirus 1 | 69.51 | 76 | dsRNA | MZ600504.1 | Fungi | unclassified Riboviria | No rank |
| 43063 | 538 | Partial | Erysiphe necator associated umbra-like virus 4 | 81.47 | 73 | ssRNA(+) | MN627470.1 | Fungi | Tombusviridae | Umbravirus |
| 24588 | 749 | Partial | Soybean leaf-associated mitovirus 2 | 88.26 | 72 | ssRNA(+) | KT598239.1 | Fungi | Mitoviridae | Mitovirus |
| 60822 | 438 | Partial | Figwort mosaic virus | 100 | 72 | dsDNA-RT | NC_003554.1 | Plant | Caulimoviridae | Caulimovirus |
| 2254 | 2600 | Complete | Botourmiaviridae sp. | 71.35 | 71 | ssRNA(+) | MZ679459.1 | Arthropods, plants, fungi | Botourmiaviridae | No rank |
| 47211 | 510 | Partial | Sclerotinia sclerotiorum botybirnavirus 5-WX1 | 73.3 | 71 | dsRNA | MT646408.1 | Fungi | unclassified Riboviria | Botybirnavirus |
| 39492 | 566 | Partial | Fusarium sambucinum mitovirus 3 | 80.58 | 67 | ssRNA(+) | LC596827.1 | Fungi | Mitoviridae | Mitovirus |
| 16460 | 950 | Partial | Agaricus bisporus virus X | 100 | 56 | ssRNA(+) | AJ421989.1 | Fungi | unclassified Riboviria | No rank |
| 5334 | 1747 | Partial | Fusarium asiaticum mitovirus 1 | 71.76 | 53 | ssRNA(+) | MW686204.1 | Fungi | Mitoviridae | Mitovirus |
| 5321 | 1748 | Partial | Plasmopara viticola lesion associated mitovirus 13 | 72.81 | 49 | ssRNA(+) | MN539774.1 | Fungi | Mitoviridae | Mitovirus |
| 21091 | 821 | Partial | Uncultured endornavirus | 98.21 | 13 | ssRNA | HE579690.1 | Plant, fungi | Endornaviridae | No rank |
| 41577 | 549 | Partial | Soybean leaf-associated ssRNA virus 2 | 97.73 | 8 | ssRNA | KT598234.1 | Plant, fungi | unclassified Riboviria | No rank |
| 5287 | 1754 | Partial | Cedratvirus lausannensis | 72.9 | ND | dsDNA | LT907979.1 | Amoeba | ND | ND |
| 40107 | 561 | Partial | Cowpox virus | 73.83 | ND | dsDNA | KY463519.1 | Vertebrates | ND | ND |

*Continued from previous page*. Supplementary Table 9. Putative viral sequence information for the Winnsboro, Louisiana library. Sequence data can be found in fasta format in Github under a file named “LA2_Louisiana-Winnsboro.” Red font indicates bacteriophage sequences. Red font with light red fill color are sequences with no significant similarity found with NCBI Blastn search. Green, blue, and gold fill colors are sequences with query coverage of 70-100%, 50-69%, and <50%, respectively. ND= not determined.

| **Contig No.** | **Length (bp)** | **Genome**  **completeness** | **Putative virus name** | **Identity (%)** | **Query cover (%)** | **Type** | **Accession** | **Known hosts** | **Family** | **Genus** |
| --- | --- | --- | --- | --- | --- | --- | --- | --- | --- | --- |
| 7730 | 599 | Partial | Soybean ilarvirus I | 80.66 | 100 | ssRNA(+) | OL539725.1 | Plants | Bromoviridae | Ilarvirus |
| 9098 | 559 | Partial | Soybean leaf-associated mitovirus 3 | 91.95 | 100 | ssRNA(+) | KT598240.1 | Fungi | Mitoviridae | Mitovirus |
| 10740 | 517 | Partial | Chicken megrivirus | 89.94 | 100 | ssRNA(+) | KF961187.1 | Vertebrates | Picornaviridae | Megrivirus |
| 11056 | 510 | Partial | Botourmiaviridae sp. | 96.08 | 100 | ssRNA(+) | NC_043521.1 | Fungi | Botourmiaviridae | No rank |
| 11609 | 498 | Partial | uncultured partitivirus | 96.39 | 100 | dsRNA | HE579647.1 | Plants, fungi | Partitiviridae | No rank |
| 13531 | 464 | Partial | Grapevine partitivirus | 90.09 | 100 | dsRNA | JX658570.1 | Plants, fungi | Partitiviridae | No rank |
| 14435 | 450 | Partial | Soybean leaf-associated mitovirus 3 | 97.56 | 100 | ssRNA(+) | KT598240.1 | Fungi | Mitoviridae | Mitovirus |
| 15183 | 439 | Partial | Soybean leaf-associated ourmiavirus 1 | 97.49 | 100 | ssRNA(+) | NC_043521.1 | Fungi | Botourmiaviridae | Scleroulivirus |
| 15730 | 432 | Partial | Tobacco ringspot virus | 97.22 | 100 | RNA | ON229939.1 | Plants | Secoviridae | Nepovirus |
| 4219 | 759 | Partial | Erysiphe necator associated ourmia-like virus 75 | 93.54 | 99 | ssRNA(+) | MN611603.1 | Fungi | Botourmiaviridae | Ourmiavirus |
| 5191 | 702 | Partial | Erysiphe necator associated ourmia-like virus 36 | 77.2 | 99 | ssRNA(+) | MN611564.1 | Fungi | Botourmiaviridae | Ourmiavirus |
| 9926 | 536 | Partial | Agaricus bisporus virus X | 97.56 | 99 | Unkn | AJ421989.1 | Fungi | unclassified viruses | No rank |
| 11032 | 510 | Partial | Beticola ourmia-like virus 3 | 92.14 | 99 | ssRNA(+) | MZ568929.1 | Fungi | Botourmiaviridae | No rank |
| 18010 | 404 | Partial | Erysiphe necator associated ourmia-like virus 75 | 96.77 | 99 | ssRNA(+) | MN611603.1 | Fungi | Botourmiaviridae | Ourmiavirus |
| 2131 | 967 | Partial | Soybean ilarvirus I | 84.29 | 98 | ssRNA(+) | OL539725.1 | Plants | Bromoviridae | Ilarvirus |
| 2 | 5762 | Complete | Cotton leafroll dwarf virus | 99.21 | 97 | ssRNA | OQ300127.1 | Plants | Solemoviridae | Polerovirus |
| 15599 | 434 | Partial | Basella alba alphaendornavirus 1 | 70.74 | 90 | ssRNA(+) | NC_043109.1 | Fungi, plants, oomycetes | Endornaviridae | Alphaendornavirus |
| 12637 | 479 | Partial | Erysiphe necator associated mitovirus 23 | 78.55 | 83 | ssRNA(+) | MN611669.1 | Fungi | Mitoviridae | Mitovirus |
| 9874 | 537 | Partial | Soybean leaf-associated mitovirus 2 | 81.4 | 64 | ssRNA(+) | KT598239.1 | Fungi | Mitoviridae | Mitovirus |

Supplementary Table 10. Putative viral sequence information for the Jackson Springs, North Carolina library. Sequence data can be found in fasta format in Github under a file named “NC3_North_Carolina-Jackson_Springs.” Green and blue fill colors are sequences with query coverage of 70-100% and 50-69%, respectively.

| **Contig No.** | **Length (bp)** | **Genome**  **completeness** | **Putative virus name** | **Identity (%)** | **Query cover (%)** | **Type** | **Accession** | **Known hosts** | **Family** | **Genus** |
| --- | --- | --- | --- | --- | --- | --- | --- | --- | --- | --- |
| 20308 | 424 | Partial | Murid betaherpesvirus 1 | 99.29 | 100 | DNA | OY639225.1 | Vertebrates | Orthoherpesviridae | Muromegalovirus |
| 15 | 6041 | Complete | Cotton leafroll dwarf virus | 99.54 | 99 | ssRNA | OQ300128.1 | Plants | Solemoviridae | Polerovirs |
| 6 | 7007 | Partial | Pseudomonas phage Misse | 72.04 | 95 | DNA | MW286267.1 | Bacteria | Bacteriophage | No rank |
| 8 | 6869 | Partial | Pseudomonas phage Strit | 72.04 | 95 | DNA | MW286267.1 | Bacteria | Bacteriophage | No rank |
| 13 | 6116 | Partial | Pseudomonas phage Strit | 72.67 | 94 | DNA | MW286268.1 | Bacteria | Bacteriophage | No rank |
| 40 | 3850 | Partial | Pseudomonas phage Strit | 78.63 | 92 | DNA | MW286268.1 | Bacteria | Bacteriophage | No rank |
| 173 | 2462 | Partial | Suillus luteus mitovirus 1 | 68.17 | 79 | RNA | OQ862545.1 | Fungi | Mitoviridae | Mitovirus |
| 4169 | 888 | Partial | Leviviridae sp. | 73.68 | 78 | ssRNA | ON162753.1 | Bacteria | Bacteriophage | No rank |
| 54 | 3613 | Partial | Pseudomonas phage Strit | 73.95 | 67 | DNA | MW286268.1 | Bacteria | Bacteriophage | No rank |
| 33 | 4164 | Partial | Pseudomonas phage Misse | 72.47 | 50 | DNA | MW286267.1 | Bacteria | Bacteriophage | No rank |
| 36 | 3967 | Partial | Pseudomonas phage Misse | 80.7 | 34 | DNA | MW286267.1 | Bacteria | Bacteriophage | No rank |
| 2460 | 1094 | Complete | Peach latent mosaic viroid | 86 | 4 | ssRNA | DQ222075.1 | Plants | Avsunviroidae | Pelamoviroid |

Supplementary Table 11. Putative viral sequence information for the Blackville, South Carolina library. Sequence data can be found in fasta format in Github under a file named “CSCR_South_Carolina-Blackville.” Red and blue font indicates bacteriophage and viroid sequences, respectively. Green, blue, and gold fill colors are sequences with query coverage of 70-100%, 50-69%, and <50%, respectively.

| **Family** | **Virus name** | **Primer set (5ʹ - 3ʹ)** | **Product size (bp)** | **Gene** | **Thermocycler conditions** | **Locations** | **Source** |
| --- | --- | --- | --- | --- | --- | --- | --- |
| *Botourmiaviridae* | Plant associated botourmia-like virus 1 | F-CCCAATTACGGTTCGGGGAA  R-TGGGTCACCGAGGAATCTCT | 540 | RdRp | - 95°C-1min   40 cycles:   - 95°C-15 sec, 62°C-20 sec, 72°C-40 sec - 72°C-3 min | Fairhope, Alabama | This study |
| *Fiersviridae* | Grapevine-associated levi-like virus 10 | F-CGATTGACCCCTTTCTGGCT  R-TGAACGTGCAAATGAGGGGA | 515 | RdRp | - 95°C-1min   40 cycles:   - 95°C-15 sec 62°C-20 sec, 72°C-40 sec, - 72°C-3 min | Brewton, Alabama | This study |
| *Mitoviridae* | Soybean leaf-associated mitovirus 2 | F-TCCTCTCATAGGCAATCCAGT  R-CCGATTTCACTCCAAAGGCG | 417 | RdRp | - 95°C-1min   40 cycles:   - 95°C-15 sec, 62°C-20 sec, 72°C-40 sec - 72°C-3 min | Prattville, Alabama;  Quincy, Florida | This study |
| *Solemoviridae* | Cotton leafroll dwarf virus | F-CCACCTAGRCGCAACAGGCGT  R-CGAGGCCTCGGAGATGAACT | 307 | Coat protein | - 95°C-1min   35 cycles:   - 95-15 sec, 62°C-20 sec, 56°C-10 sec, 72°C-40 sec,   72°C-5 min   - 72°C-3 min | Fairhope, Alabama;  Headland, Alabama;  Prattville, Alabama;  Brewton, Alabama | Sharman et al. (2015)^44^ |

Supplementary Table 12: Primers and RT-PCR conditions used in this study that successfully yielded the expected amplicons and high-quality Sanger sequences. For every reaction, RNA was first converted to cDNA following manufacturer’s instructions. Bp= base pairs, RdRp= RNA-dependent RNA-polymerase. Primers for grapevine partitivirus are not included due to amplification and Sanger sequencing results inconsistency.

| **Virus name** | **Primer name** | **Primer set (5ʹ - 3ʹ)** | **Product size (bp)** | **Gene** | **Thermocycler conditions** | **Sample location/library** | **RT-PCR product obtained** |
| --- | --- | --- | --- | --- | --- | --- | --- |
| Cassava vein mosaic virus | 41-42Vir | F-TCACCAAAGGTCTGCTCGTC  R-ATGATGCGTAGTGACCCGAC | 674 | N/A | 95°C-2min  35 cycles:  95-1min 42-62°C-0.5 min 72°C-0.5 min  72°C-5 min  4°C-Indef. | Fairhope, AL | No |
|  | 43-44Vir | F-CTCCATGGCTCAGACCACTC  R-CATCAAGGGCATCGTAGGCT | 452 |  |  |  | No |
| Cotton leafroll dwarf virus | CLRDV3675F  Pol3982R | F-CCACCTAGRCGCAACAGGCGT  R-CGAGGCCTCGGAGATGAACT | 307 | Coat protein | 95°C-1min  35 cycles:  95-15 seg 62°C-20 sec 56°C-10 sec 72°C-40 sec  72°C-5 min  72°C-3 min  4°C – Indef. | Fairhope, AL; Headland, AL; Prattville, AL; Brewton, AL | Yes |
| Plant associated botourmia-like virus 1 | 45-46Vir | F-ATGAAGGCAGTTCGGGTCTG  R-TCCTCGCAAAGAAGCCCATT | 493 | RdRp | 95°C-1min  40 cycles:  95-15 seg 62°C_42°C-20 sec 72°C-40 sec  72°C-2 min  4°C – Indef. | Fairhope, AL | No |
|  | 47-48Vir | F-CCCAATTACGGTTCGGGGAA  R-TGGGTCACCGAGGAATCTCT | 540 |  | 95°C-1min  40 cycles:  95-15 seg 62°C-20 sec 72°C-40 sec  72°C-2 min  4°C – Indef. |  | Yes |
| Erysiphe necator associated narnavirus 31 | 49-50Vir | F-CTCCTGCAAAGGAGTCCCTG  R-CACGATCCATCCGTCTCCAG | 669 | N/A | 95°C-1min  40 cycles:  95-15 seg 62°C_42°C-20 sec 72°C-40 sec  72°C-2 min  4°C – Indef. | Prattville, AL | No |
|  | 51-52Vir | F-CTTTTATCGGGCACAGGGGT  R-TACGTACGAGCCTCACGGTA | 517 |  |  |  | No |
| Cladosporium cladosporioides negative-stranded RNA virus 1 | 53-54Vir | F-GGGCTCCCAATTGCCTTACT  R-TCTTTTCGACTGTCACCGGG | 509 | N/A | 95°C-1min  40 cycles:  95-15 seg 62°C_42°C-20 sec 72°C-40 sec  72°C-2 min  4°C – Indef. | Headland, AL | No |
|  | 55-56Vir | F-GGGGTTAGTCGACAGCAACA  R-TGGAGATAGCTCGTCCACCA | 628 |  | 95°C-1min  40 cycles:  95-15 seg 50°C-20 sec 72°C-40 sec  72°C-2 min  4°C – Indef. |  | No |
| Grapevine-associated levi-like virus 10 | 61-62Vir | F-CGTTGCAGTTTTCGCTGTGA  R-ACGGGAACACGTGGCATAAT | 432 | RdRp | 95°C-1min  40 cycles:  95-15 seg 62°C_42°C-20 sec 72°C-40 sec  72°C-2 min  4°C – Indef. | Brewton, AL | Yes |
|  | 63-64Vir | F-CGATTGACCCCTTTCTGGCT  R-TGAACGTGCAAATGAGGGGA | 515 |  | 95°C-1min  40 cycles:  95-15 seg 62°C-20 sec 72°C-40 sec  72°C-2 min  4°C – Indef. |  | Yes |
| Alternaria arborescens mitovirus 1 | 65-66Vir | F-GCTGGTCCTGTTGGTAAAGC  R-ATGCACCCATAGGTTGACCC | 541 | RdRp | 95°C-1min  40 cycles:  95-15 seg 54°C-20 sec 72°C-40 sec  72°C-2 min  4°C – Indef. | Winnsboro, LA | No |
|  | 67-68Vir | F-GGGTCAACCTATGGGTGCAT  R-ACATCACCTGAAGGGATCACA | 588 |  | 95°C-1min  40 cycles:  95-15 seg 54°C-20 sec 72°C-40 sec  72°C-2 min  4°C – Indef. |  | No |
| Aphid lethal paralysis virus | 69-70Vir | F-CCAATGTCGCCTGAATGGGA  R-TGTCCAACCACGAATCAGCA | 403 | RdRp | 95°C-1min  40 cycles:  95-15 seg 62°C_42°C-20 sec 72°C-40 sec  72°C-2 min  4°C – Indef. | Tifton, GA | No |
|  | 71-72Vir | F-TCGCGAACCACTCTGACTTG  R-TCGTACCCAGTTTGGTGCAT | 452 |  |  |  | No |
| Grapevine partitivirus | 73-74Vir | F-TGAACGGGAAGAGTTCGCAA  R-TCCGGAAAGCACTGTCATCC | 426 | RdRp | 95°C-1min  40 cycles:  95-15 seg 57°C_48°C-15 sec 72°C-40 sec  72°C-2 min  4°C – Indef. | Headland, AL, Brewton, AL, Quincy, FL,Jackson Springs, NC | No |
|  | 75-76Vir | F-ATGGTTTCACTCGTCGTCCC  R-ATCGGAGGCTTCAGACATGC | 389 |  | 95°C-1min  40 cycles:  95-15 seg 46°C-20 sec 72°C-40 sec  72°C-2 min  4°C – Indef. |  | Yes |
| Potato yellow dwarf nucleorhabdovirus | 77-78Vir | F-ATGATCAACCCTCGTCTCGG  R-CTCGTCTTGTCCACCCTTGA | 353 | RdRp | 95°C-1min  40 cycles:  95-15 seg 62°C_42°C-20 sec 72°C-40 sec  72°C-2 min  4°C – Indef. | Prattville, AL | No |
|  | 79-80Vir | F-GGGGTCCGGGTATAGTGAAT  R-CTCACCTGAGCACAGGCTTT | 368 |  |  |  | No |
| Soybean leaf-associated mitovirus 2 | 81-82Vir | F-TGCACACCAGTGCACAACTA  R-TGCTTGAGAGAAGGGTTTTCCT | 457 | RdRp | 95°C-1min  40 cycles:  95-15 seg 62°C_42°C-20 sec 72°C-40 sec  72°C-2 min  4°C – Indef. | Prattville, AL; Quincy, FL | No |
|  | 83-84Vir | F-TCCTCTCATAGGCAATCCAGT  R-CCGATTTCACTCCAAAGGCG | 417 |  | 95°C-1min  40 cycles:  95-15 seg 62°C-20 sec 72°C-40 sec  72°C-2 min  4°C – Indef. |  | Yes |
| Peanut mottle virus | 85-86Vir | F-CAACCAACCTTGCTTGCCAT  R-TCCCTCTTTCACCCCAGACA | 414 | Peptidase | 95°C-1min  40 cycles:  95-15 seg 43°C-20 sec 72°C-40 sec  72°C-2 min  4°C – Indef. | Jay, FL | No |
|  | 87-88Vir | F-TGTTGGCTTGGCGTTTTCAA  R-GAATGCCAATGGTTGGCGG | 305 | Coat Protein | 95°C-1min  40 cycles:  95-15 seg 62°C_42°C-20 sec 72°C-40 sec  72°C-2 min  4°C – Indef. |  | No |

*Continued from previous page.* Supplementary Table 13. List of selected sequences used for RT-PCR validation in this study. Primers for cotton leafroll dwarf virus were previously published by Sharman et al. (2015)^44^. Primers and RT-PCR conditions that successfully yielded the expected amplicons are presented in Supplementary Table 12. Sanger sequencing was not successful from grapevine partitivirus using primer set 75-76Vir.


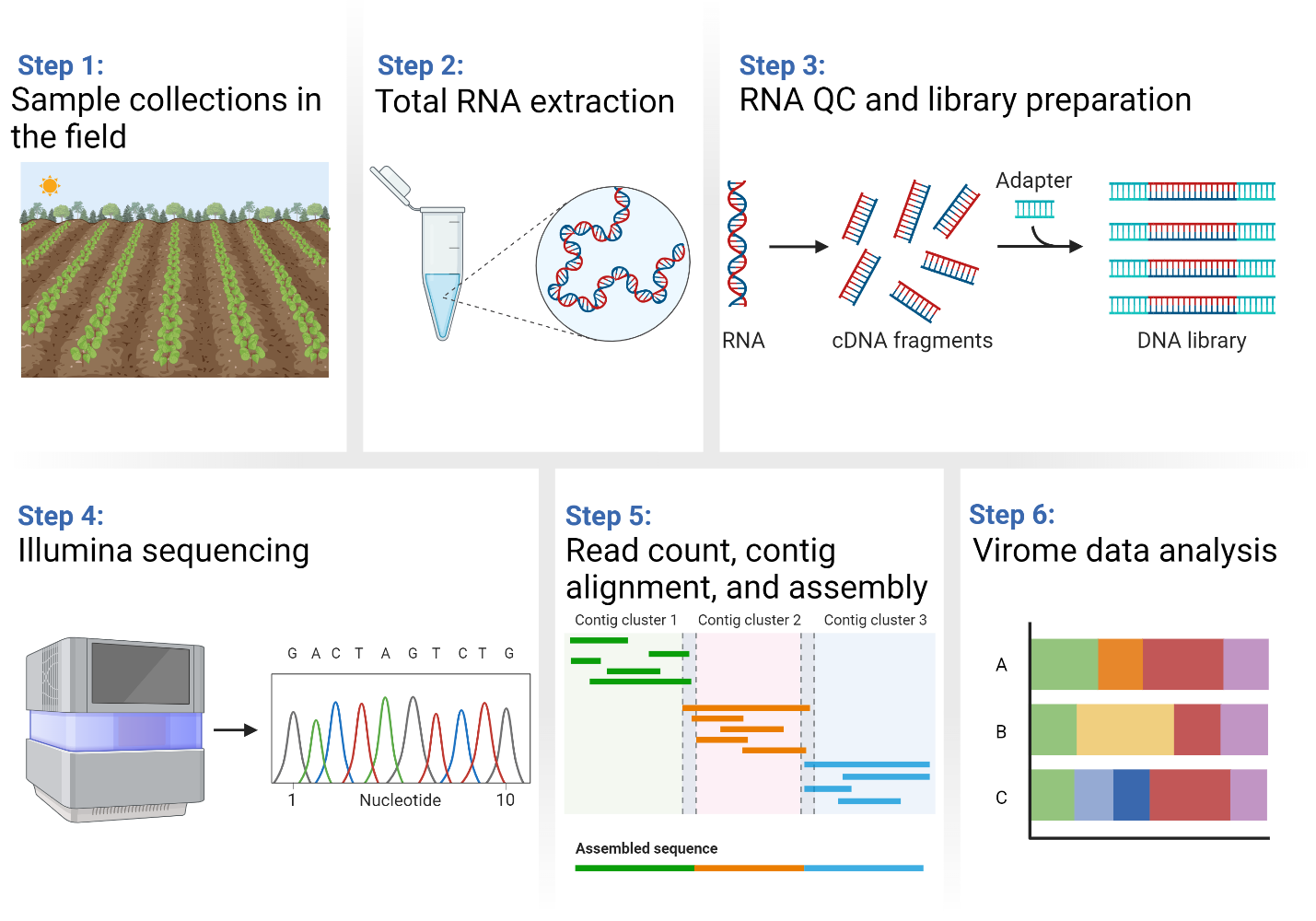


Supplementary Figure 1. Illustration of steps for the RNA-sequencing and virome analysis conducted in this investigation. Diagram created in BioRender.com.

Supplementary Figure 2. Locations across the southern United States where cotton samples were collected to test for the presence of putative viral sequences. Map was created using R software version 2025.05.1+513.
